# Calibrated simplex-mapping classification

**DOI:** 10.1371/journal.pone.0279876

**Published:** 2023-01-17

**Authors:** Raoul Heese, Jochen Schmid, Michał Walczak, Michael Bortz

**Affiliations:** 1 Fraunhofer Center for Machine Learning, Kaiserslautern, Germany; 2 Fraunhofer Institute for Industrial Mathematics ITWM, Kaiserslautern, Germany; Shandong Normal University, CHINA

## Abstract

We propose a novel methodology for general multi-class classification in arbitrary feature spaces, which results in a potentially well-calibrated classifier. Calibrated classifiers are important in many applications because, in addition to the prediction of mere class labels, they also yield a confidence level for each of their predictions. In essence, the training of our classifier proceeds in two steps. In a first step, the training data is represented in a latent space whose geometry is induced by a regular (*n* − 1)-dimensional simplex, *n* being the number of classes. We design this representation in such a way that it well reflects the feature space distances of the datapoints to their own- and foreign-class neighbors. In a second step, the latent space representation of the training data is extended to the whole feature space by fitting a regression model to the transformed data. With this latent-space representation, our calibrated classifier is readily defined. We rigorously establish its core theoretical properties and benchmark its prediction and calibration properties by means of various synthetic and real-world data sets from different application domains.

## 1 Introduction

In many classification tasks, it is not sufficient to merely predict the class label $\hat{y}(x)$ for a given feature space point *x*. Instead, it is often important to also have good predictions $\hat{p}(y|x)$ for the class label probabilities, because these probability predictions provide a measure for the confidence one can have in the individual class label predictions $\hat{y}(x)$. Such additional confidence information is important in many applications, for instance in clinical applications [1]. Classifiers that come with such additional class probability predictions are called calibrated. Some classifiers from methods like logistic regression or Gaussian process classification (GPC) are intrinsically calibrated. Also, there are various methods to calibrate an intrinsically non-calibrated classifier or to improve the calibration quality of an ill-calibrated classifier [2–5].

### 1.1 Contribution

In this paper, we propose a novel supervised learning method for multi-class classification that yields classifiers with a high potential to be intrinsically well-calibrated. It can be applied to general classification problems in an arbitrary metrizable feature space X of possibly non-numeric features and with an arbitrary number *n* of classes with labels $y\in Y≔\{l_{1},\ldots ,l_{n}\}$. Starting from a training data set

$$\begin{array}{c} D≔\{\left(x_{i},y_{i}\right):i\in \left\{1,\ldots ,D\right\}\}\subset X\times Y \end{array}$$ (1)

of feature space points $x_{1},\ldots ,x_{D}\in X$ together with associated class labels $y_{i}=y(x_{i})\in Y$, the training of our classifier proceeds in two training steps:

1. In a first step, the training datapoints *x*_1_, …, *x*_*D*_ are transformed by means of a suitable training data transformation $f:\{x_{1},\ldots ,x_{D}\}\to Z$ to a latent space Z, which we partition into *n* cone segments $C_{l_{1}},\ldots ,C_{l_{n}}$ corresponding to the *n* classes in D and defined in terms of a regular (*n* − 1)-dimensional simplex in Z.
2. In a second step, a regression model $\hat{f}:X\to Z$ is trained based on the transformed training datapoints (*x*_1_, *f*(*x*_1_)), …, (*x*_*D*_, (*f*(*x*_*D*_)), which are obtained by means of the training data transformation *f* from the first step. In this manner, the latent space representation of the training data is extended to the whole feature space.

We design the training data transformation *f* such that the latent space counterpart *f*(*x*_*i*_) of each datapoint *x*_*i*_ is located in the corresponding cone segment $C_{y_{i}}=C_{y(x_{i})}$ and such that the location of *f*(*x*_*i*_) in this segment reflects the distances of *x*_*i*_ from its own-class and its foreign-class datapoint neighbors. Concerning the choice of the distance metric and the number of neighbors used in the definition of *f*, we are completely free, and the same is true for the choice of the regression model $\hat{f}$. In particular, these quantities can be freely customized and tuned to the particular problem at hand.

As soon as the above training steps have been performed, our classifier $\hat{y}:X\to Y$ is readily obtained. Indeed, its class label prediction $\hat{y}(x)$ for a given feature space point x∈X is the label of the (first) cone segment $C_{y}$ that contains $\hat{f}(x)$, the regression model’s point prediction for *x*. If in addition to these point predictions, the regression model also provides probabilistic predictions, then our classifier yields predictions $\hat{p}(y|x)$ for the class label probabilities as well. Specifically, these class label probability predictions read

$$\begin{array}{c} \hat{p}\left(y|x\right)≔\int_{C_{y}}\hat{q}\left(z|x\right)\;dz\;\left(y\in Y\right), \end{array}$$ (2)

where $\hat{q}(\;\cdot \;|x)$ for a given x∈X is the regression model’s prediction for the probabilistic distribution of latent space points. As a classifier coming with class label probability predictions, our classifier is calibrated. We refer to it as a *calibrated simplex-mapping classifier* (CASIMAC) because the underlying latent-space mapping *f* is defined in terms of the vertices of a simplex in Z.

We point out that the concept of leveraging Bayesian probabilistic prediction power combined with latent space mappings has been studied before. Several recent publications propose to couple a deep neural network with Gaussian processes (GPs) for an improved uncertainty estimate of model predictions [6–10]. Alternative approaches explore the use of deep neural networks not as feature extraction methods but, for instance, to suitably estimate the mean functions of GPs [11] or to predict their covariance functions and hyperparameters [12]. Yet, due to the high complexity of the deep neural network components in these models, the algorithms mentioned above are well-suited for large data sets with abundant training data available [13]. In the present paper, by contrast, we propose a simple latent space representation of the original feature space as the core component of a well-calibrated classifier that also works on less complex data sets. In particular, our method has recently been successfully used for an industrial application [14].

In summary, our contribution consists of the following parts:

1. We propose a novel supervised learning method for multi-class classification with a simplex-like latent space.
2. We rigorously establish the theoretical background including detailed proofs.
3. We find that the computational effort of making predictions with our proposed classifier is comparatively low (in contrast to, e. g., GPC).
4. We show how the latent space of our proposed classifier can be suitably visualized.
5. We benchmark the prediction and calibration properties of our proposed classifier.

Additionally, we discuss potential use cases and further research directions.

### 1.2 Simple example

**Fig 1:**
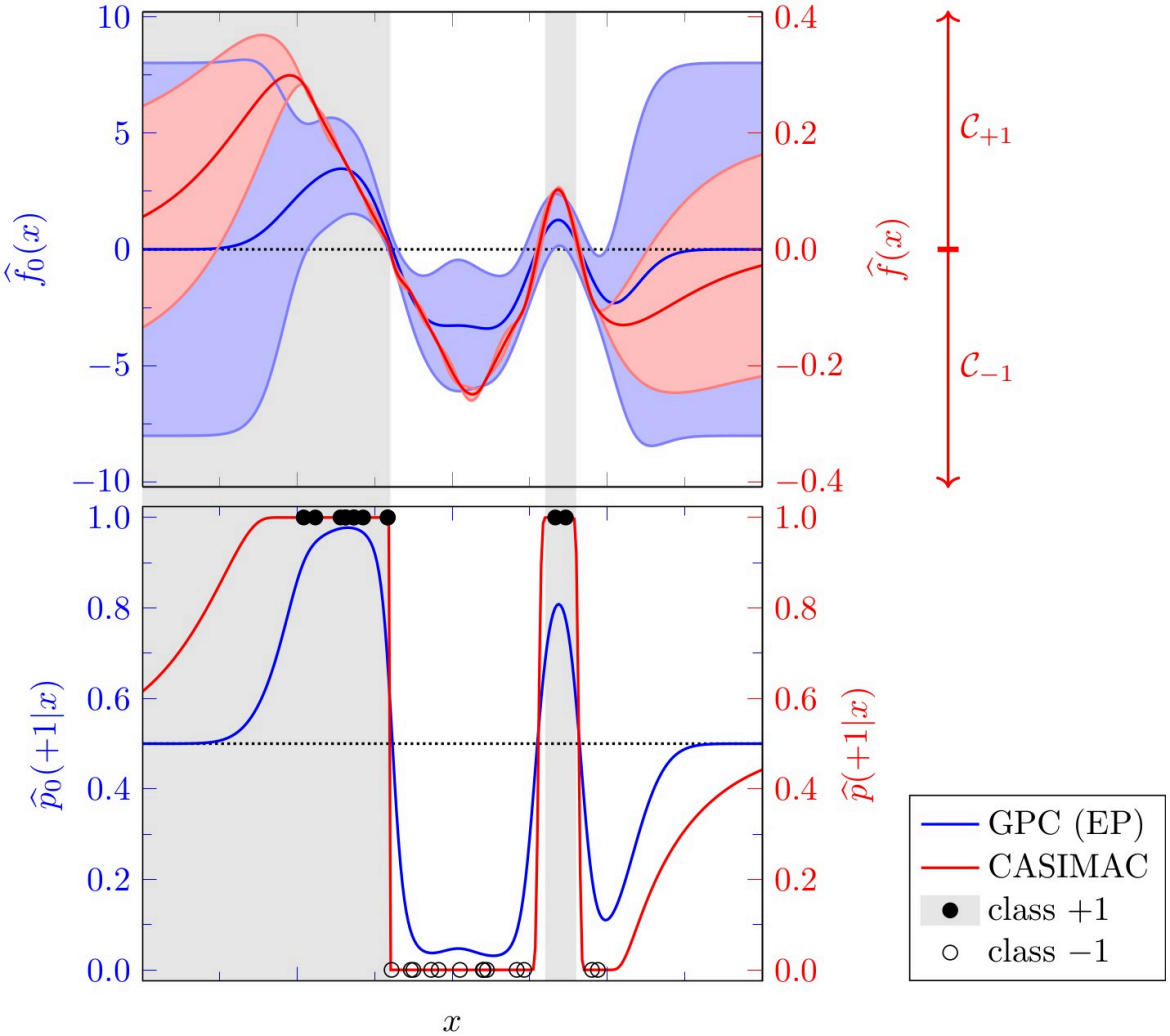
Exemplary binary classification problem. An exemplary binary classification problem with one-dimensional feature space X=R and class labels *y* ∈ {−1, +1}. We compare a GPC (using a radial-basis-function kernel and expectation propagation (EP) with the implementation from [19]) and our CASIMAC (based on a GPR with Matérn kernel as the underlying regression model), both trained on the same *D* = 25 training datapoints. The top plot shows the expectation values ${\hat{f}}_{0}(x)$, $\hat{f}(x)$ and the standard deviations of the latent space distributions ${\hat{q}}_{0}(\;\cdot \;|x)$, $\hat{q}(\;\cdot \;|x)$ for both approaches (on two different scales), whereas the bottom plot contains the class probability predictions ${\hat{p}}_{0}(+1|x)$ and $\hat{p}(+1|x)$ for both approaches. The gray and white areas indicate the true regions of the class + 1 and −1, respectively. All datapoints are sampled without noise from these regions. Finally, the dotted horizontal lines (⋅⋅⋅⋅⋅⋅⋅⋅⋅⋅⋅⋅⋅) represent the decision boundaries. The corresponding cone segments for CASIMAC, $C_{-1}=(-\infty ,0]$ and $C_{+1}=[0,\infty )$, are shown in the top plot.

In order to concretize the aforementioned assets of our method and paint a more intuitive picture, we briefly discuss a simple case with *n* = 2 classes (i. e., a binary classification problem), which is shown in Fig 1. The two class labels are chosen as *l*_1_ ≔ −1 and *l*_2_ ≔ + 1, respectively. In that case, our latent space Z=R is just the real line and the cone segments simply are $C_{-1}=(-\infty ,0]$ and $C_{+1}=[0,\infty )$, the negative and the positive half-axis, respectively. Consequently, our definition of the class label predictions simplifies to

$$\begin{array}{c} \hat{y}\left(x\right)=-1\;\text{if}\;\hat{f}\left(x\right)\leq 0\;\text{and}\;\hat{y}\left(x\right)=+1\;\text{if}\;\hat{f}\left(x\right)>0, \end{array}$$ (3)

while our definition of the class probability predictions simplifies to

$$\begin{array}{c} \hat{p}\left(-1|x\right)=\int_{-\infty }^{0}\hat{q}\left(z|x\right)\;dz=1-\hat{p}\left(+1|x\right)\;\text{and}\;\hat{p}\left(+1|x\right)=\int_{0}^{\infty }\hat{q}\left(z|x\right)\;dz. \end{array}$$ (4)

As before, $\hat{f}$ is a regression model that is fitted to the transformed training datapoints (*x*_1_, *f*(*x*_1_)), …, (*x*_*D*_, *f*(*x*_*D*_)) and $\hat{q}(\;\cdot \;|x)$ is the associated predictive a posteriori distribution for *x* based on the same transformed training datapoints.

A simple choice for the regression model $\hat{f}$ is a Gaussian process regressor (GPR). And a possible choice for the training data transformation *f* on which $\hat{f}$ depends is the simple distance-based map defined as follows:

$$\begin{array}{c} f\left(x_{i}\right)≔\text{signed}\;\text{distance}\;\text{of}\;x_{i}\;\text{from}\;\text{its}\;\text{closest}\;\text{opposite-class}\;\text{datapoint}\;\text{neighbor}, \end{array}$$ (5)

where the sign in this formula is simply given by the class label $y_{i}=y(x_{i})\in Y=\{\pm 1\}$ of the datapoint *x*_*i*_ and the distance is to be understood w.r.t. the chosen metric on the feature space X. If we train a CASIMAC with these choices for *f* and $\hat{f}$ on an exemplary training data set with *D* = 25 points in the one-dimensional feature space X=R, we obtain the class probability predictions $\hat{p}(+1|x)$ depicted in red in Fig 1 (bottom plot). If we train a GPC on the same training data, we obtain the respective class probability predictions ${\hat{p}}_{0}(+1|x)$ depicted in blue. As usual,

$$\begin{array}{c} {\hat{p}}_{0}\left(+1|x\right)=\int_{-\infty }^{\infty }\sigma \left(z\right)\cdot {\hat{q}}_{0}\left(z|x\right)\;dz, \end{array}$$ (6)

where *σ*(*z*) ≔ 1/(1 + e^−*z*^) represents the logistic sigmoid function and ${\hat{q}}_{0}(\;\cdot \;|x)$ is the predictive a posteriori distribution of the GPC for the test point *x* [15, 16].

We immediately see from the class probability plots in Fig 1 that our CASIMAC has a high confidence in its class label predictions in regions of densely sampled datapoints, as one would intuitively expect. In contrast, the GPC has considerably less confidence in its class label predictions at or near the datapoints despite its high prediction accuracy. In essence, this is because the training data transformation *f* that underlies our classifier takes into account the actual distances of the datapoints and because the latent space probability density $\hat{q}(\;\cdot \;|x)$ is considerably more concentrated around its expectation value $\hat{f}(x)$ than is the case for its analog ${\hat{q}}_{0}(\;\cdot \;|x)$. Another downside of the GPC—further impairing its calibration quality—is that its class probability predictions ${\hat{p}}_{0}(+1|x)$ can be computed only approximately. In fact, already the computation of the non-normal distribution ${\hat{q}}_{0}(\;\cdot \;|x)$, a *D*-dimensional integral, requires quite sophisticated approximations like the Laplace approximation [15], expectation propagation [15], variational inference [17], or the Markov chain Monte Carlo approximation [18], to name a few. As opposed to this, no approximations are required for the computation of the CASIMAC class probabilities $\hat{p}(+1|x)$ because there is a closed-form expression for them, due to the normality of the distributions $\hat{q}(\;\cdot \;|x)$ assumed in our example from Fig 1.

### 1.3 Structure of the paper

In section 2, we formally introduce our CASIMAC method. We explain in detail the two training steps as well as how predictions of a trained classifier can be obtained. We also show how our latent space mappings can be leveraged for the convenient visualization of inter- or intra-class relationships in the data, especially in the case of a high-dimensional feature space and a moderate number of classes. In Section 3, we apply our method to various data sets and compare its performance and calibration qualities to several well-established benchmark classifiers. In particular, we demonstrate that our approach can be applied to many different application domains because our training data transformation reflects actual distances in the data set and because the respective distance metric as well as the regression model are freely customizable. Section 4 concludes the paper with a summary and an outlook on possible future research. The appendix collects the mathematical and technical background underlying our CASIMAC method. In S1 Appendix, we rigorously prove the results needed for an in-depth and mathematically sound understanding of the method. In S2 Appendix, in turn, we summarize the main features of our implementation, and S3 Appendix summarizes how the hyperparameters of the models in Section 3 were chosen.

## 2 Calibrated simplex-mapping classification

In this section, we formally introduce our proposed calibrated simplex-mapping classifier, briefly referred to as CASIMAC. Sections 2.1 and 2.2 explain in detail the two training steps outlined in Section 1.1. In Section 2.3, we give the precise definition of our CASIMAC and, moreover, we explain how its predictions $\hat{y}(x)$ and $\hat{p}(y|x)$ for the class labels and the class label probabilities can be calculated in a computationally favorable manner. In Section 2.4 we finally explain how the latent space representation upon which our classification method relies can also be exploited for visualization purposes. Here and in the following, we consistently denote the training data set and the training datapoints as in (1) and write

$$\begin{array}{c} y\left(x_{i}\right)≔y_{i} \end{array}$$ (7)

for the true class labels of the training datapoints *x*_1_, …, *x*_*D*_. Also, X always denotes the feature space—which is only assumed to be metrizable and, in particular, need not be embedded in any $R^{m}$—and Y denotes the set of class labels. As usual, a set is called metrizable iff there exists at least one metric on it [20]. We see below that we actually only need so-called semimetrics [21] and we exploit this even greater flexibility in our last application example. If X is embedded in some $R^{m}$, then of course infinitely many metrics exist on X, namely at least all the metrics induced by the *ℓ*^*p*^-norms ‖⋅‖_*p*_ on $R^{m}$ for *p* ∈ [1, ∞) ∪ {∞}. In order to reduce cumbersome double indices, we assume from here on that the class labels are just 1, …, *n* (instead of the general labels *l*_1_, …, *l*_*n*_). In short, we assume that

$$\begin{array}{c} Y≔\{1,\ldots ,n\} \end{array}$$ (8)

without loss of generality.

### 2.1 Training data transformation to a latent space

In the first training step of our method, we transform the feature space training datapoints *x*_1_, …, *x*_*D*_ by means of a suitably designed training data transformation

$$\begin{array}{c} f:D_{X}\to Z\;\left(D_{X}≔\left\{x_{1},\ldots ,x_{D}\right\}\right) \end{array}$$ (9)

to a suitable latent space Z. We choose this latent space to be $Z≔R^{n-1}$, where *n* ≥ 2 as before is the number of classes observed in the training data D. We decompose this space into *n* conically shaped segments $C_{1},\ldots ,C_{n}$, which are defined by the vertices *p*_1_, …, *p*_*n*_ of a regular (*n* − 1)-dimensional simplex S in Z having barycenter 0 and being at unit distance from their barycenter 0, that is,

$$\begin{array}{c} \sum_{i\in Y}\;p_{i}=0\;\text{and}\;{\left\|p_{i}\right\|}_{2}=1\;\left(i\in Y\right). \end{array}$$ (10)

See S1 Appendix (Proposition 2). Specifically, we define the segment $C_{k}$ as the cone that is spanned by the mirrored vertices −*p*_*i*_ with *i* ≠ *k*, that is,

$$\begin{array}{c} C_{k}≔\{z\in Z:z=\sum_{i\in Y\backslash \{k\}}c_{i}\cdot \left(-p_{i}\right)\;\text{for}\;\text{some}\;c_{i}\in \left[0,\infty \right)\}. \end{array}$$ (11)

In view of (10.a), the vertex *p*_*k*_ lies on the central ray of $C_{k}$, which is why we also refer to *p*_*k*_ as the central vector of $C_{k}$. Also, the segments $C_{1},\ldots ,C_{n}$ cover the whole latent space Z and any two of these segments overlap only at their boundaries. In short,

$$\begin{array}{c} \underset{k\in Y}{⋃}\;C_{k}=Z\;\text{and}\;C_{k}^{\circ }\cap C_{l}=\emptyset \;\left(k\neq l\right), \end{array}$$ (12)

where $C_{k}^{\circ }$ is the interior of $C_{k}$, that is, the set $C_{k}$ without its boundary [20]. It is given by

$$\begin{array}{c} C_{k}^{\circ }=\{z\in Z:z=\sum_{i\in Y\backslash \{k\}}c_{i}\cdot \left(-p_{i}\right)\;\text{for}\;\text{some}\;c_{i}\in \left(0,\infty \right)\}. \end{array}$$ (13)

**Fig 2:**
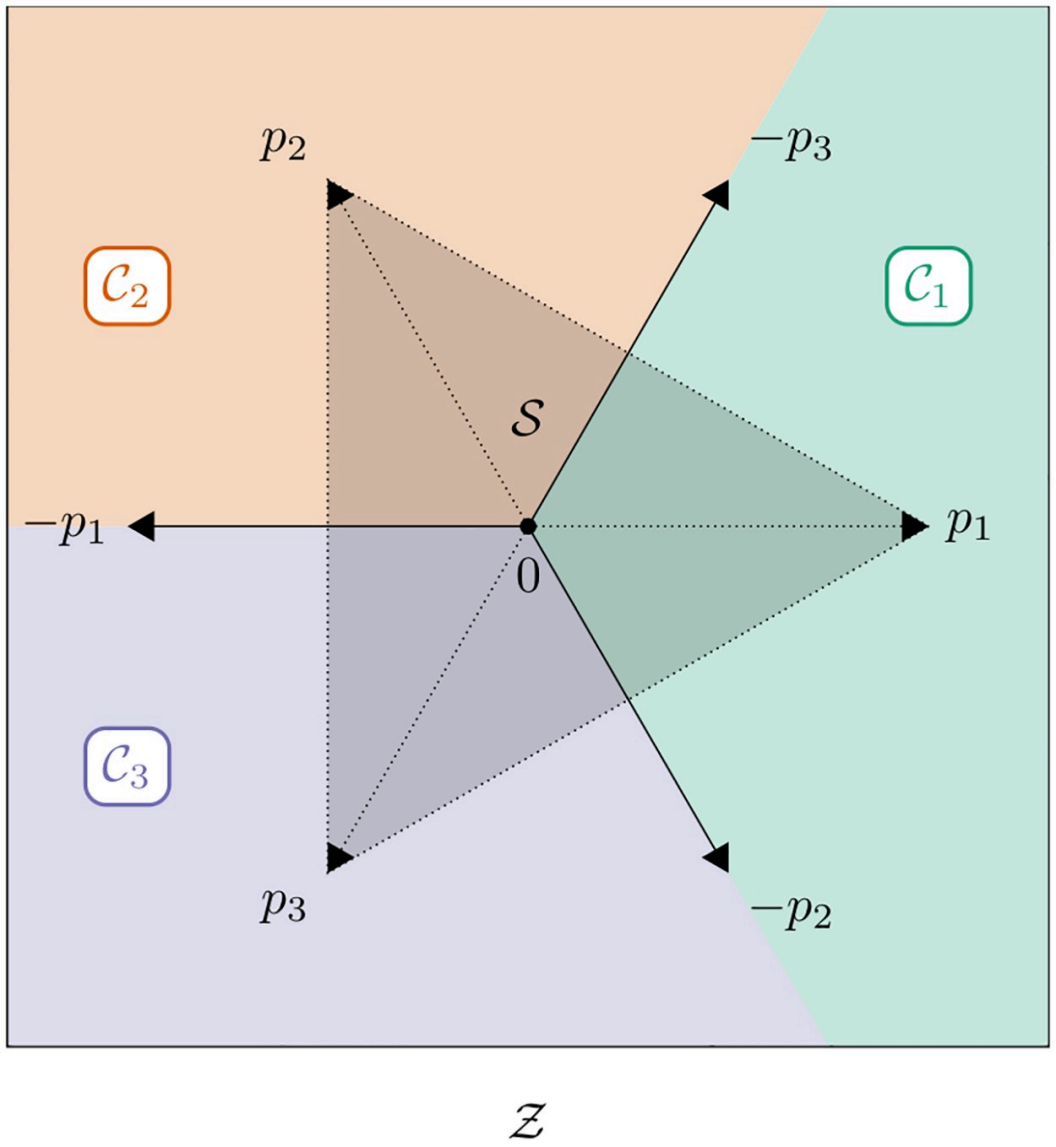
Segmentation of the latent space. Segmentation of the latent space $Z=R^{2}$ of a ternary classification problem (*n* = 3) into three congruent cone segments $C_{1},C_{2},C_{3}$, each associated with one of the three classes as indicated by the respective colors. The vertices *p*_1_, *p*_2_, *p*_3_ of the simplex S (marked by the gray shading) with barycenter 0 lie on the central ray of the respective segments. The borders of the segments are defined by the vertices −*p*_1_, −*p*_2_, −*p*_3_ of the mirrored simplex −S.

And finally, the segments $C_{1},\ldots ,C_{n}$ are pairwise congruent. All these statements are proven rigorously in S1 Appendix (Lemma 5 and Propositions 6 and 7). In the special case of just two or three classes, they can also be easily verified graphically. See Fig 2, for instance.

With the help of the above segmentation of the latent space Z, we can define the training data transformation (9). We design this mapping *f* such that it maps each training datapoint $x\in D_{X}$ to the cone segment $C_{y(x)}$ corresponding to its class label *y*(*x*) in such a way that the location of *f*(*x*) in this cone segment $C_{y(x)}$ reflects the distances of *x* from its own-class and from its foreign-class neighbors. We choose the location of *f*(*x*) based on the following premises:

1. *f*(*x*) should be located the farther in the direction of *p*_*y*(*x*)_ (and thus the farther inside the cone segment $C_{y(x)}$), the closer *x* is to its class-*y*(*x*) datapoint neighbors
2. *f*(*x*) should be located the farther in the direction of −*p*_*y*_ (and thus the farther away from the cone segment $C_{y}$), the farther *x* is away from its class-*y* datapoint neighbors for y∈Y\{y(x)}.

Specifically, we define *f* as follows:

$$\begin{array}{c} f\left(x\right)=f_{\alpha ,\beta ,k_{\alpha },k_{\beta },d}\left(x\right)≔\alpha A_{k_{\alpha },d}\left(x\right)\cdot p_{y(x)}+\sum_{y\in Y\backslash \{y(x)\}}\beta R_{k_{\beta },d}\left(x,y\right)\cdot \left(-p_{y}\right) \end{array}$$ (14)

for every training datapoint $x\in D_{X}$. In particular, the coefficient $A_{k_{\alpha },d}(x)$ indicates how far *f*(*x*) is pulled into the own-class segment $C_{y(x)}$, while the coefficient $R_{k_{\beta },d}(x,y)$ indicates how far *f*(*x*) is pulled away from the foreign-class segment $C_{y}$ for y∈Y\{y(x)}. We therefore refer to these coefficients as the attraction and the repulsion coefficients of *x* and we define them, as indicated above, in terms of the mean distance of *x* from its *k*_*α*_ closest datapoint neighbors of its own class *y*(*x*) or, respectively, from its *k*_*β*_ closest datapoint neighbors of the foreign class y∈Y\{y(x)}. That is,

$$\begin{array}{c} A_{k_{\alpha },d}\left(x\right)≔{\left({\text{NN}}_{k_{\alpha },d}\left(x,D_{X,y(x)}\right)\right)}^{-1}\;\text{and}\;R_{k_{\beta },d}\left(x,y\right)≔{\text{NN}}_{k_{\beta },d}\left(x,D_{X,y}\right), \end{array}$$ (15)

where $D_{X,y}≔\{x_{i}\in D_{X}:y_{i}=y(x_{i})=y\}$ is the set of all training datapoints belonging to class *y* and

$$\begin{array}{c} {\text{NN}}_{k,d}\left(x,X\right)≔\underset{X^{\prime }\subset X\backslash \left\{x\right\}\;\text{with}\;\left|X^{\prime }\right|=k}{\text{min}}\frac{1}{k}\sum_{x^{\prime }\in X^{\prime }}d\left(x,x^{\prime }\right) \end{array}$$ (16)

is the mean distance of *x* from its *k* nearest neighbors from the subset X⊂X, the distance being measured in terms of some arbitrary semimetric [21]

$$\begin{array}{c} d:X\times X\to [0,\infty ) \end{array}$$ (17)

on the feature space X, that is, *d*(*x*, *x*′) = *d*(*x*′, *x*) for all $x,x^{\prime }\in X$ (symmetry) and *d*(*x*, *x*′) = 0 if and only if *x* = *x*′. At least one such semimetric exists on X because this space was assumed to be semimetrizable at the beginning of Section 2. Also, *α*, *β* ∈ [0, ∞) and $k_{\alpha },k_{\beta }\in N$ are user-defined hyperparameters satisfying

$$\begin{array}{c} \alpha +\beta >0\;\text{and}\;k_{\alpha }\leq c-1\;\text{and}\;k_{\beta }\leq c, \end{array}$$ (18)

where $c≔\text{min}\{|D_{X,y}|:y\in Y\}$ is the cardinality of the smallest training data class. Conditions (18.b) and (18.c) guarantee that all the attraction and repulsion coefficients are strictly positive finite real numbers, that is,

$$\begin{array}{c} 0<A_{k_{\alpha },d}\left(x\right),R_{k_{\beta },d}\left(x,y\right)<\infty \end{array}$$ (19)

for all $x\in D_{X}$ and all y∈Y\{y(x)} (Proposition 10). In most of our applications, *d* is a proper metric, that is, a semimetric that also satisfies the triangle inequality. In our last application example, though, we make explicit use of semimetrics as well.

Since the domain $D_{X}$ of *f* and the attraction and repulsion coefficients occurring in the definition of *f* obviously depend on the training data set D, so does the training data transformation *f* itself, and we sometimes make this dependence explicit by writing

$$\begin{array}{c} f\left(x\right)=f\left(x|D\right)=f_{\alpha ,\beta ,k_{\alpha },k_{\beta },d}\left(x|D\right). \end{array}$$ (20)

**Fig 3:**
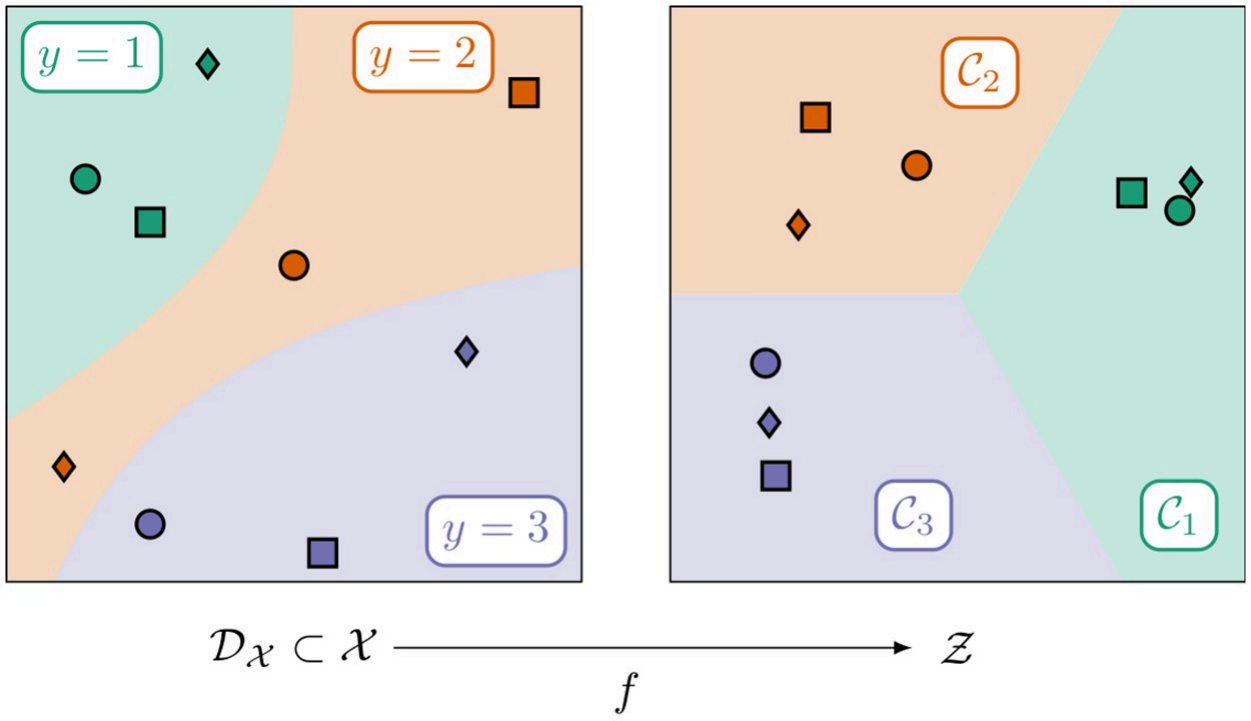
Illustration of the training data transformation. Illustration of the training data transformation $f:\{x_{1},\ldots ,x_{D}\}\to Z=R^{2}$ from (14) for an exemplary ternary classification problem (*n* = 3) with a two-dimensional feature space $X\subset R^{2}$. The colored regions on the left illustrate the true regions of the three classes with their respective labels, whereas the points denote the (noiselessly) sampled training datapoints *x*_1_, …, *x*_*D*_. We sample three points from each class (i. e., *D* = 9) and use different symbols (∘, ◽ and ♢) to uniquely identify each point. As the distance metric *d* underlying *f*, we choose the Euclidean distance. The other hyperparameters are chosen as *α* ≔ 0, *β* ≔ 1 and *k*_*α*_, *k*_*β*_ ≔ 1, respectively. The figure shows that the farther a training datapoint *x*_*i*_ (for *i* ∈ {1, …, *D*}) is away from its nearest foreign-class neighbor, the clearer is the membership of *f*(*x*_*i*_) to the respective cone segment $C_{y(x_{i})}$.

It is straightforward to verify that the training data transformation *f* from (14), with *α* ≔ 0, *β* ≔ 1, *k*_*α*_, *k*_*β*_ ≔ 1, reduces to the mapping from (5) in the special case of just *n* = 2 classes. It is also easy to verify from (14), using the barycenter condition (10.a) along with (13), (18.a) and (19), that

$$\begin{array}{c} f\left(x\right)=\sum_{y\in Y\backslash \{y(x)\}}(\alpha A_{k_{\alpha },d}\left(x\right)+\beta R_{k_{\beta },d}\left(x,y\right))\cdot \left(-p_{y}\right)\in C_{y(x)}^{\circ } \end{array}$$ (21)

for every $x\in D_{X}$ (Proposition 10). In other words, *f*(*x*) for every training datapoint $x\in D_{X}$ lies in the interior $C_{y(x)}^{\circ }$ of the corresponding cone segment. Specifically, the membership of *f*(*x*) to $C_{y(x)}^{\circ }$ is the clearer, the closer *x* is to its *k*_*α*_ nearest own-class neighbors and the farther *x* is away from its *k*_*β*_ nearest foreign-class neighbors. Fig 3 illustrates this behavior of the training data transformation *f* for the simple case of a ternary classification problem (*n* = 3).

We point out that this behavior of *f* is generic in the sense that it is independent of the number of classes and independent of the specific choices of *α*, *β*, *k*_*α*_, *k*_*β*_ and *d*. In particular, we can freely choose and tune the semimetric *d* as well as the hyperparameters *α*, *β*, *k*_*α*_, *k*_*β*_ within the bounds (18) to the specific classification problem at hand [22]. We make ample use of this customization flexibility for the exemplary classification problems in Section 3.

### 2.2 Training of a regression model based on the transformed data

In the second training step of our method, we train a regression model

$$\begin{array}{c} \hat{f}:X\to Z \end{array}$$ (22)

from the feature space X to the latent space Z. As the training data set for this regression model, we take the transformed training data set

$$\begin{array}{c} D^{f}≔\left\{\left(x_{i},f\left(x_{i}\right)\right):i\in \left\{1,\ldots ,D\right\}\right\}\subset X\times Z \end{array}$$ (23)

consisting of the feature space training datapoints $x_{1},\ldots ,x_{D}\in D_{X}$ together with their latent space counterparts $f(x_{1}),\ldots ,f(x_{D})\in Z$ which are obtained by means of the training data transformation *f* from the first training step as defined in (14).

**Fig 4:**
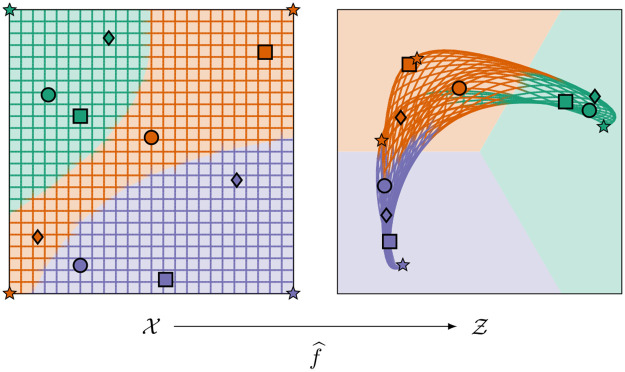
Illustration of the regression model. Illustration of the regression model $\hat{f}:X\to Z=R^{2}$ for the exemplary ternary classification problem from Fig 3. We choose a GPR model with a Matérn kernel, which is fitted to the transformed training datapoints (*x*_1_, *f*(*x*_1_),, …, (*x*_*D*_, *f*(*x*_*D*_)) that are obtained from the training data transformation *f* according to Fig 3. In order to illustrate the effect of $\hat{f}$, we show how the points of the rectangular grid on the left get transformed by $\hat{f}$. In particular, we show this transformation for the corner points marked by stars. The color of any point *x* on the grid and, respectively, of any point $\hat{f}(x)$ on the transformed grid indicates the true class of *x*.

In sharp contrast to *f*, the regression model $\hat{f}$ is defined on the whole of X (instead of only the training datapoints *x*_1_, …, *x*_*D*_) and thus yields latent space predictions for arbitrary feature space points x∈X, especially for hitherto unobserved and unclassified feature space points. Fig 4 illustrates the effect of the regression model for the special case of a ternary classification problem (*n* = 3).

Since both $D_{X}$ and f=f(⋅|D) depend on the original training data set D, so does the regression model $\hat{f}$ and we sometimes make this dependence explicit by writing

$$\begin{array}{c} \hat{f}\left(x\right)=\hat{f}\left(x|D\right). \end{array}$$ (24)

We point out that—similarly to the choice of the hyperparameters *α*, *β*, *k*_*α*_, *k*_*β*_ and *d* of the training data transformation—the specific choice of the regression model is completely arbitrary.

In particular, we can choose a suitable probabilistic regression model that is able to not only predict a single latent space point $\hat{f}(x)=\hat{f}(x|D)$ but also an entire probability density $\hat{q}(\;\cdot \;|x)=\hat{q}(\;\cdot \;|D,x)$ for every x∈X. Such a so-called predictive a posteriori distribution indicates the distribution of the latent space points for given *x* and a given data set D. In particular, the predictive a posteriori distribution $\hat{q}(\;\cdot \;|x)$ indicates the level of confidence that the model has in its point prediction $\hat{f}(x)$, namely it is all the more confident in the point prediction $\hat{f}(x)$ the more $\hat{q}(\;\cdot \;|x)$ is concentrated around $\hat{f}(x)$. A typical example for regression models which provide both a point prediction $\hat{f}(x)$ and a probability-density prediction $\hat{q}(\;\cdot \;|x)$ for every x∈X are GPR models [15, 16]. As is well-known, for these models the point predictions $\hat{f}(x)$ are completely determined by the predictive a posteriori distribution $\hat{q}(\;\cdot \;|x)$, namely as the maximum a posteriori prediction, that is,

$$\begin{array}{c} \hat{f}\left(x\right)=\underset{z\in Z}{\text{argmax}}\;\hat{q}\left(z|x\right). \end{array}$$ (25)

We make ample use of GPR models in our benchmark examples from Section 3.

### 2.3 Calibrated simplex-mapping classifier

After the two training steps explained above have been performed, we can define and put to use our CASIMAC. We define it as the classifier

$$\begin{array}{c} \hat{y}:X\to Y \end{array}$$ (26)

that assigns to each feature space point x∈X the label *y* of the first cone segment $C_{y}$ that contains $\hat{f}(x)$. That is,

$$\begin{array}{c} \hat{y}\left(x\right)≔\hat{g}\left(\hat{f}\left(x\right)\right)≔\text{min}\left\{y\in Y:\hat{f}\left(x\right)\in C_{y}\right\}, \end{array}$$ (27)

where $\hat{f}(x)$ is the regression model’s latent space prediction for *x* and where

$$\begin{array}{c} \hat{g}\left(z\right)≔\text{min}\left\{y\in Y:z\in C_{y}\right\} \end{array}$$ (28)

is the label of the first cone segment containing *z*. Since by (12.a) every latent space point z∈Z is contained in at least one of the segments $C_{1},\ldots ,C_{n}$, the expression (27) really yields a well-defined classifier. Since, on the other hand, the segments overlap at their respective borders, a given latent space point z∈Z is contained in several segments if it is located exactly on such a border. In this special case, we need to decide for one of the overlapping segments, to obtain a classifier that assigns a single label (instead of multiple labels) to each feature space point. In our definition (27), we decide for the first of the segments containing $z=\hat{f}(x)$ for the sake of simplicity, hence the minimum in that formula.

In view of the dependence of the regression model $\hat{f}=\hat{f}(\;\cdot \;|D)$ on the training data set D, our CASIMAC depends on D as well and, to emphasize this, we sometimes write

$$\begin{array}{c} \hat{y}\left(x\right)=\hat{y}\left(x|D\right)=\hat{g}\left(\hat{f}\left(x|D\right)\right). \end{array}$$ (29)

**Fig 5:**
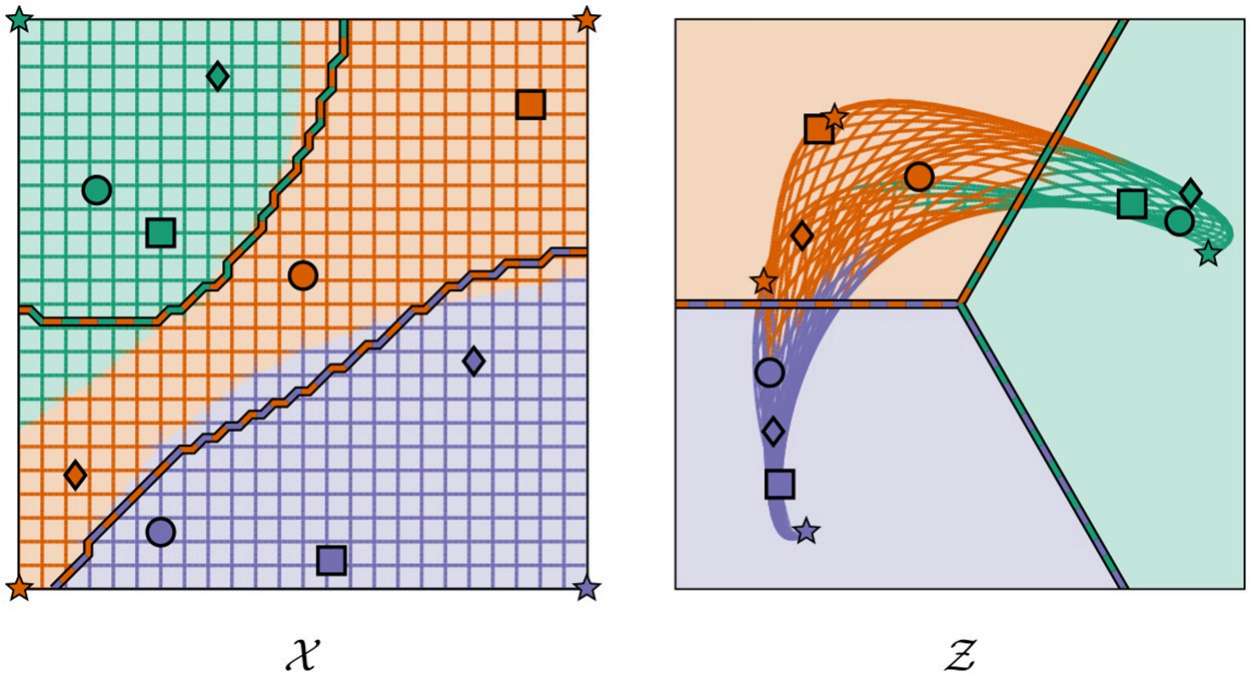
Illustration of our proposed calibrated simplex-mapping classifier. Illustration of our proposed calibrated simplex-mapping classifier $\hat{y}:X\to Y$ for the exemplary ternary classification problem from Fig 3 based on the GPR model $\hat{f}$ from Fig 4. According to our definition (27), the classifier determines for each feature space point x∈X (on the left) the cone segment containing the latent space counterpart $\hat{f}(x)\in Z$ (on the right) and then takes the label of this cone segment as the class label prediction $\hat{y}(x)\in Y$ for *x*. In other words, the cuts of the cone segment borders through the latent space Z determine the class membership of each data point *x* according to its learned latent space position $\hat{f}(x)$. We mark the predicted class boundaries (on the left) as well as the corresponding cone segment boundaries (on the right) by dashed lines. As can be seen, the predicted class boundaries in X do not deviate much from the true class boundaries indicated by the different background and grid colors on the left. In other words, our classifier produces only a few misclassifications. This can also be seen from the fact that the color of most of the transformed grid points in Z on the right (indicating the true class) is identical to the underlying background color (indicating the predicted class).

According to the definition (27), in order to practically compute the class label prediction $\hat{y}(x)$ for a given feature space point x∈X, we have to determine all cone segments that contain $\hat{f}(x)$. Yet, in view of the purely geometric definition (11) of the cone segments, it is not so clear at first glance how this can be done in a computationally feasible and favorable manner—especially in the case of many classes (*n* ≥ 5) where the segments cannot be (directly) visualized anymore. We find, however, that the segments containing $\hat{f}(x)$ can be determined solely in terms of the distances

$$\begin{array}{c} \|\hat{f}\left(x\right)-p_{1}{\|}_{2},\ldots ,\|\hat{f}\left(x\right)-p_{n}{\|}_{2} \end{array}$$ (30)

of $\hat{f}(x)$ from the central vectors *p*_1_, …, *p*_*n*_ of the segments $C_{1},\ldots ,C_{n}$. Specifically, we prove in S1 Appendix (Theorem 8) that a latent space point z∈Z belongs to the segment $C_{y}$ if and only if

$$\begin{array}{c} {\left\|z-p_{y}\right\|}_{2}=\underset{l\in Y}{\text{min}}{\left\|z-p_{l}\right\|}_{2}, \end{array}$$ (31)

that is, if and only if its distance from the other segments’ central vectors *p*_*l*_, *l* ≠ *y*, is at least as large as its distance from the central vector *p*_*y*_ of $C_{y}$. Consequently, the geometrically inspired definition (27) of our CASIMAC can be recast in the form

$$\begin{array}{c} \hat{y}\left(x\right)=\text{min}\{y\in Y:\|\hat{f}\left(x\right)-p_{y}{\|}_{2}=\underset{l\in Y}{\text{min}}\|\hat{f}\left(x\right)-p_{l}{\|}_{2}\} \end{array}$$ (32)

(Corollary 11). Computationally, (32) is much easier to evaluate than (27) since $\hat{y}(x)$ can be determined simply by calculating all distances (30) and by then choosing the smallest one. In our CASIMAC implementation, we therefore use (32) to perform predictions. We illustrate the method in Fig 5.

If the regression model $\hat{f}$ perfectly fits the training data (23) in the sense that

$$\begin{array}{c} \hat{f}\left(x_{i}\right)=f\left(x_{i}\right)\;\left(i\in \left\{1,\ldots ,D\right\}\right), \end{array}$$ (33)

then it is easy to see from (27), using (12.b) and (21), that

$$\begin{array}{c} \hat{y}\left(x_{i}\right)=y\left(x_{i}\right)=y_{i}\;\left(i\in \left\{1,\ldots ,D\right\}\right) \end{array}$$ (34)

(Corollary 11). In other words, if the regression model is perfectly interpolating in the sense of (33), our CASIMAC perfectly reconstructs the true class labels of the training datapoints. A typical example of perfectly interpolating regression models are noise-free GPR models [15, 16].

If the regression model apart from its point predictions also provides probabilistic predictions, we can estimate the class probabilities for arbitrary feature space points *x*. In that case, there is a latent probability density $\hat{q}(\;\cdot \;|D,x)$ on Z for every x∈X which on the one hand determines the point prediction $\hat{f}(x)$ in some way—for instance, as the maximum a posteriori estimate (25)—and which on the other hand also determines the uncertainty of this prediction. Since the regression model $\hat{f}$ is our model for observations of latent-space points, the associated probabilities $\hat{q}(z|D,x)$ can be considered as estimates for the probability of observing the latent-space point z∈Z for a given x∈X. Since, moreover, $\hat{y}(x)=y$ if and only if $\hat{f}(x)\in \{z\in Z:\hat{g}(z)=y\}$ by the definition (27) of our classifier, the expression

$$\begin{array}{c} \hat{p}\left(y|D,x\right)≔\int_{\left\{\hat{g}=y\right\}}\hat{q}\left(z|D,x\right)\;dz=\int_{Z}I_{\left\{\hat{g}=y\right\}}\left(z\right)\cdot \hat{q}\left(z|D,x\right)\;dz \end{array}$$ (35)

is an estimate for the probability of observing class *y* for *x*. We therefore take this estimate as our classifier’s prediction for the probability of class *y* given *x*. In the above equation, $I_{\left\{\hat{g}=y\right\}}$ denotes the indicator function

$$\begin{array}{c} I_{\left\{\hat{g}=y\right\}}\left(z\right)=\{\begin{array}{c} 1,\;\text{if}\;\hat{g}\left(z\right)=y\\ 0,\;\text{otherwise} \end{array} \end{array}$$ (36)

with respect to the set $\left\{\hat{g}=y\}≔\{z\in Z:\hat{g}(z)=y\right\}$. In view of (12.b) and (28), we have $C_{y}^{\circ }\subset \{\hat{g}=y\}\subset C_{y}$ for every y∈Y and thus

$$\begin{array}{c} \hat{p}\left(y|D,x\right)=\int_{C_{y}}\hat{q}\left(z|D,x\right)\;dz=\int_{C_{y}^{\circ }}\hat{q}\left(z|D,x\right)\;dz \end{array}$$ (37)

are alternative expressions for our classifier’s class probability prediction (Corollary 12).

If the probability densities $\hat{q}(\;\cdot \;|D,x)$ belong to a normal distribution and the number of classes is *n* = 2, then the class probability predictions (37) can be computed by means of a simple analytical formula, namely (118) in S1 Appendix (Proposition 13). If, by contrast, the probability densities $\hat{q}(\;\cdot \;|D,x)$ do not belong to a normal distribution or the number of classes is *n* > 2, then the multi-dimensional integrals in (37) can still be computed approximately. In our CASIMAC implementation, we use Monte Carlo sampling for these approximate computations of the class probability predictions (37) for *n* > 2. See (119) in S1 Appendix (Proposition 13). Standard examples of regression models $\hat{f}$ with normally distributed probability densities $\hat{q}(\;\cdot \;|D,x)$ are provided by GPR models. In particular, the class probabilities for the binary classification problem from Fig 1 were evaluated analytically.

### 2.4 Visualization

Apart from its central use in the definition of our classifier, the latent space representation given by *f* and $\hat{f}$ can also be beneficially used for visualization purposes.

In particular, the training data transformation *f* can be exploited for visually detecting inter- or intra-class relationships in the data. Inspecting the latent space representation from Fig 3, for instance, we find that the transformed datapoints of class 1 and 3 are farther apart from each other than they both are apart from the transformed datapoints of class 2. We could have directly seen that, of course, by inspecting the untransformed datapoints in feature space, which is only 2-dimensional here. After all, class 2 lies between class 1 and 3, as the left panel of Fig 3 reveals. Such a direct inspection of the data in feature space, however, is possible only for feature spaces with small dimensions. In case the feature space is high-dimensional or not even embedded in an $R^{m}$, though, we can still use its latent space representation to uncover relations between the datapoints. All we need for that is a latent space of moderate dimension *n* − 1, ideally *n* ≤ 4.

Since our latent space $Z=R^{n-1}$ is an unbounded set by definition, the latent space counterparts *f*(*x*_1_), …, *f*(*x*_*D*_) of the datapoints can be very far apart from each other. It can therefore be useful to further transform the latent space—and with it, the points *f*(*x*_1_), …, *f*(*x*_*D*_)—to a fixed bounded reference set. A natural way of achieving this is to compress the latent space Z to the interior of the reference simplex S underlying our CASIMAC. Indeed, there is a bijective compression map

$$\begin{array}{c} C:Z\to S^{\circ } \end{array}$$ (38)

which is diffeomorphic (infinitely differentiable with infinitely differentiable inverse) and which leaves the cone segments $C_{1},\ldots ,C_{n}$ invariant in the sense that, for every z∈Z and every k∈Y,

$$\begin{array}{c} C\left(z\right)\in C_{k}\;\text{if}\;\text{and}\;\text{only}\;\text{if}\;z\in C_{k}. \end{array}$$ (39)

See S1 Appendix (Proposition 15). We use this compression map for the visualization of one of our real-world data sets below.

## 3 Benchmark

In this section, we apply our CASIMAC method to various data sets and compare its performance to several well-established benchmark classifiers. Specifically, we perform benchmarks using synthetic data in Section 3.1 and real-world data in Section 3.2. Furthermore, we exemplarily illustrate the latent space visualization in Section 3.3. In these first three subsections, the regression model underlying our classifier is a GPR. In Section 3.4, by contrast, we use a neural network as our regression model, thereby demonstrating how a more complex regression model allows us to solve a classification task with more training data. For all calculations, we use the implementation of our method [23], which is briefly outlined in S2 Appendix.

### 3.1 Synthetic data

**Fig 6:**
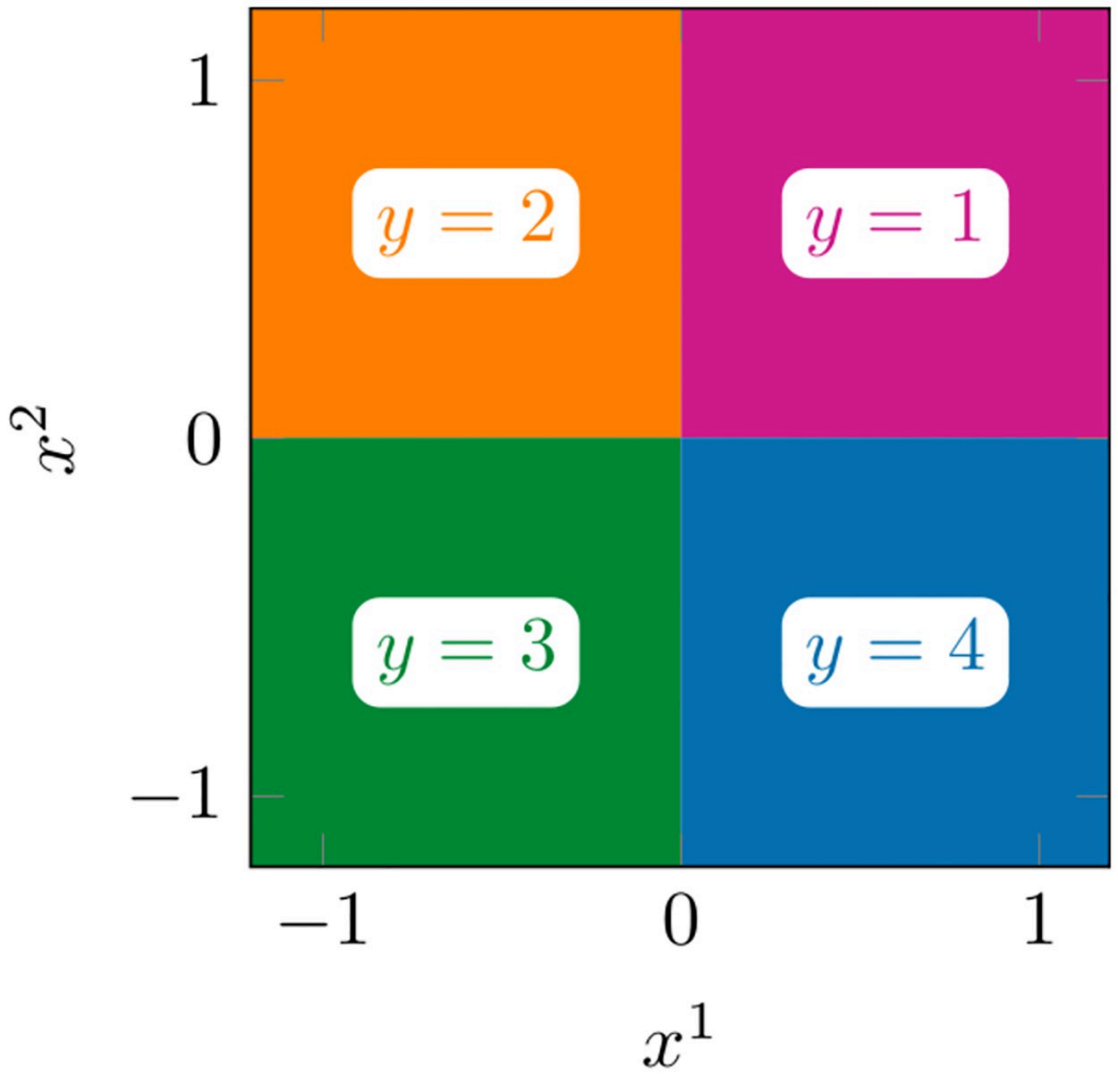
Synthetic data visualization. Illustration of the ground truth (40) for the synthetic data class labels. Synthetic data visualization.

As a first benchmark, we test our method on a synthetically generated data set. Specifically, we consider the synthetic classification problem with *n* = 4 classes and the 2-dimensional feature space $X≔[-1,1{]}^{2}$, which is based on the explicit class membership rule

$$\begin{array}{c} y_{\text{true}}\left(x\right)≔\{\begin{array}{c} 1,\;\text{if}\;x^{1}\geq 0\;\text{and}\;x^{2}\geq 0\\ 2,\;\text{if}\;x^{1}<0\;\text{and}\;x^{2}\geq 0\\ 3,\;\text{if}\;x^{1}<0\;\text{and}\;x^{2}<0\\ 4,\;\text{if}\;x^{1}\geq 0\;\text{and}\;x^{2}<0 \end{array} \end{array}$$ (40)

for features $x≔(x^{1},x^{2})\in X$. The problem is illustrated in Fig 6. In order to obtain our training data set (1), we uniformly sample *D* = 40 points from X and associate them with their true class labels according to (40). In particular, the synthetic data is free of noise. Analogously, we obtain the test data set

$$\begin{array}{c} T≔\{\left(x_{i},y_{i}\right):i\in \left\{D+1,\ldots ,D+T\right\}\} \end{array}$$ (41)

by uniformly sampling *T* = 10000 points from X and by associating them with their true class labels (40). Here and in the following, we always standardize the features based on the training data before feeding it to the considered classifiers.

As the distance metric *d* in the training data transformation (14) underlying our CASIMAC, we choose the Euclidean distance on $X\subset R^{2}$, that is,

$$\begin{array}{c} d\left(x,x^{\prime }\right)≔{\left\|x-x^{\prime }\right\|}_{2} \end{array}$$ (42)

for all $x,x^{\prime }\in X$. Also, we parameterize the weights *α*, *β* for the attraction and repulsion coefficients (15) by a single parameter *γ* ∈ [0, 1], namely

$$\begin{array}{c} \gamma ≔\beta ≔1-\alpha , \end{array}$$ (43)

so that *α*, *β* ∈ [0, 1]. And as the regression model (22) underlying our CASIMAC, we choose a GPR with a combination of a Matérn and a white-noise kernel [15]. We compare our classifier to a (one-versus-rest) GPC with the same type of kernel. The hyperparameters of both classifiers are tuned by cross-validating the training data over a pre-defined set of different setups as outlined in S3 Appendix. We made use of scikit-sklearn [24] to realize the standard models.

In total, we perform 10 classification tasks, each with different test and training data sets. For each, we use the test data set to determine the accuracy (fraction of correctly predicted points) and the log-loss (that is, the logistic regression loss or cross-entropy loss). In addition to that, we calculate the proba-loss, which we define as the mean predicted probability error

$$\begin{array}{c} \delta p≔1-\frac{1}{T}\sum_{i=D+1}^{D+T}\hat{p}\left(y=y_{\text{true}}\left(x_{i}\right)|D,x_{i}\right), \end{array}$$ (44)

where $\hat{p}(y|D,x)$ is our CASIMAC’s or the GPC’s class probability prediction, respectively. Clearly, *δp* ∈ [0, 1] with 0 being the best possible outcome and 1 the worst. We can compute this score only because we know the true class membership rule (40) underlying the problem.

The results are shown in Table 1. Clearly, our method has a better proba-loss and log-loss than the GPC, whereas the latter has a slightly better accuracy.

**Table 1:** Synthetic data test scores. Test scores for the synthetic data set based on *T* = 10000 uniformely sampled test datapoints. The proba-loss is defined in (44). We show the means and the corresponding standard deviations (in brackets) over all 10 classification tasks. The best mean results are highlighted in bold.

Score	CASIMAC	GPC
proba-loss	**0.106 (14)**	0.552(26)
log-loss	**0.406(332)**	0.825(55)
accuracy	0.913(17)	**0.924(18)**

**Fig 7:**
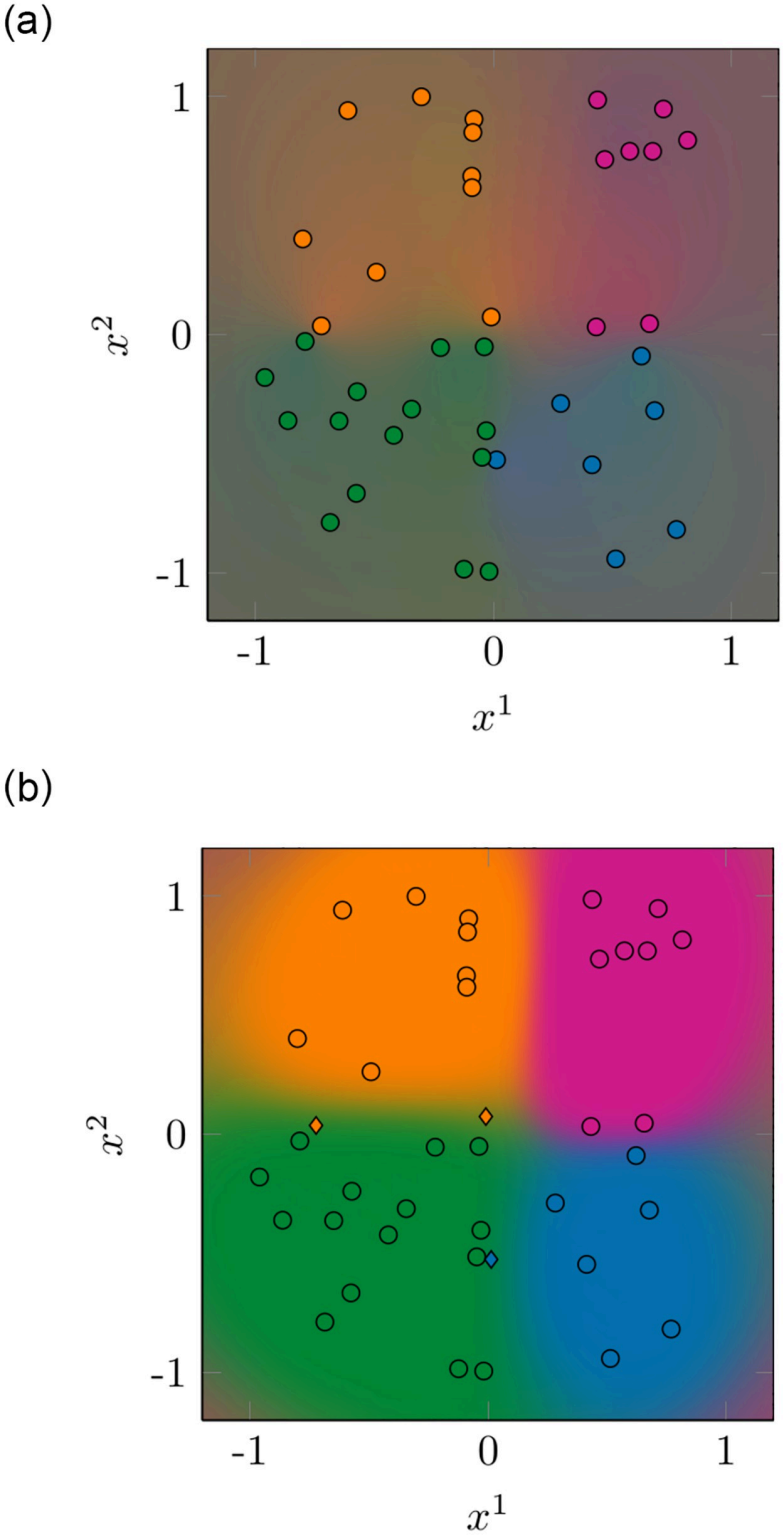
Synthetic data class probability predictions. (a) CASIMAC class probabilities. (b) GPC class probabilities. Class probability predictions of CASIMAC and of GPC for the synthetic data set. The color of each background point corresponds to the weighted average of the class colors from Fig 6 with a weight corresponding to the predicted probability of the respective class at this point. Thus, clear colors as in (a) represent high probabilities for a single class, whereas washed-out colors as in (b) represent almost uniform probabilities. We also show the training data set (consisting of *D* = 40 points) on which the classifiers have been trained. The color of the points corresponds to their true class. While most of the training datapoints are correctly classified (shown as ∘), our CASIMAC incorrectly predicts three training datapoints (shown as ♢) close to the class borders.

We also show an exemplary visualization of the predicted class probabilities in Fig 7 for a single training data set. The background colors represent a weighted average of the class colors from Fig 6 with a weight corresponding to the predicted probability of the respective class. So, for a perfectly calibrated classifier, the colors in Fig 7 would be the same as the colors in Fig 6. Although our CASIMAC has a slightly lower training accuracy than the GPC (0.925 as opposed to 1.000), it clearly exhibits brighter, more distinguishable colors. Consequently, the predicted probabilities are less uniform and correspond to a clearer decision for one of the four classes instead of an uncertain mixture. This observation corresponds to the lower proba-loss and log-loss results in Table 1.

### 3.2 Real-world data

Following the benchmark on synthetic data, we continue with a benchmark on five real-world data sets from different fields of application. Specifically, we consider the data sets from Table 2. All of these data sets are publicly available online.

**Table 2:** Data set overview. Overview of the five real-world data sets and their basic characteristics: *n* is the number of classes, *m* is the number of features or, in other words, the dimension of the feature space $X\subset R^{m}$, *I* is the total number of datapoints, and *D* is the number of training datapoints. The number *T* of test datapoints is just *T* ≔ *I* − *D*. We abbreviate the references as a) [25], b) [26], c) [27], d) [28], e) [29], f) [30], g) [2], and h) [31]. Also, we turned the originally multi-class data set pine into a binary problem as described in [2].

Name	*n*	*m*	*I*	*D*	Refs.
alcohol	5	10	125	50	a), b)
climate	2	18	540	50	a), c)
hiv	2	160	6590	125	a), d)
pine	2	200	21025	2000	e), f), g)
wifi	4	7	2000	500	a), h)

As the distance metric *d* in the training data transformation (14) underlying our CASIMAC, we choose the Euclidean distance (42) on $X\subset R^{m}$ for the data sets alcohol, climate, hiv, and pine, whereas for the remaining data set wifi we choose the taxicab distance on $X\subset R^{m}$ defined by

$$\begin{array}{c} d\left(x,x^{\prime }\right)≔{\left\|x-x^{\prime }\right\|}_{1}, \end{array}$$ (45)

because it leads to a better performance there. Additionally, we parameterize the weights *α*, *β* of the attraction and repulsion coefficients (15) as in (43) for all our data sets. And as the regression model (22) underlying our CASIMAC, we again choose a GPR with a combination of a Matérn and a white-noise kernel for all our data sets. We compare our CASIMAC on each data set to three other classifiers, namely to a GPC as before and, in addition to that, to an artificial neural network with fully-connected layers (MLP) and to a *k*-nearest neighbor classifier (kNN). This choice of classifiers was dictated by their reported good calibration properties [2]. Again, we tune the hyperparameters of the classifiers by cross-validation as outlined in S3 Appendix. As in the previous section, we made use of scikit-sklearn [24] to realize the standard models.

The test-training split of the data is performed by means of a stratified random sampling with respect to the class labels. Average test scores over 10 classification tasks with different training data sets are reported in Table 3. Note that for multi-class data sets, the f1 score is calculated as the weighted arithmetic mean over harmonic means [32], where the weight is determined by the number of true instances for each class. Analogously, we calculate the precision score (ratio of true positives to the sum of true positives and false positives) and recall score (ratio of true positives to the sum of true positives and false negatives) as weighted averages over all classes.

**Table 3:** Real-world data test scores. Test scores for the benchmarked classifiers on the five real-world data sets from Table 2. We show the means and the corresponding standard deviations (in brackets) over all 10 classification tasks. The best mean results are highlighted in bold.

Score	CASIMAC	GPC	kNN	MLP
alcohol				
accuracy	**0.907(62)**	0.897(44)	0.545(88)	0.848(47)
f1	**0.901(67)**	0.894(48)	0.526(93)	0.845(53)
precision	**0.928(44)**	0.922(30)	0.556(106)	0.872(43)
recall	**0.907(62)**	0.897(44)	0.545(88)	0.848(47)
log-loss	**0.394(338)**	0.932(50)	1.752(1442)	0.755(412)
climate				
accuracy	0.914(2)	**0.915(4)**	**0.915(1)**	0.906(12)
f1	0.877(7)	0.879(8)	0.875(2)	**0.883(15)**
precision	0.854(30)	0.861(30)	0.854(29)	**0.874(32)**
recall	0.914(2)	**0.915(4)**	**0.915(1)**	0.906(12)
log-loss	**0.249(17)**	0.281(9)	1.060(337)	0.341(48)
hiv				
accuracy	**0.902(4)**	0.900(5)	0.868(7)	0.880(6)
f1	0.898(5)	**0.898(5)**	0.862(7)	0.880(6)
precision	**0.899(4)**	0.898(5)	0.861(7)	0.880(6)
recall	**0.902(4)**	0.900(5)	0.868(7)	0.880(6)
log-loss	**0.263(17)**	0.290(7)	0.405(31)	0.404(18)
pine				
accuracy	**0.937(1)**	0.934(2)	0.916(2)	0.923(5)
f1	**0.934(1)**	0.931(2)	0.914(3)	0.922(5)
precision	**0.933(2)**	0.930(2)	0.913(3)	0.922(5)
recall	**0.937(1)**	0.934(2)	0.916(2)	0.923(5)
log-loss	**0.169(7)**	0.182(4)	0.396(117)	0.212(43)
wifi				
accuracy	**0.977(2)**	0.976(2)	0.968(3)	0.969(4)
f1	**0.977(2)**	0.976(2)	0.969(3)	0.969(4)
precision	**0.978(2)**	0.977(2)	0.969(3)	0.969(4)
recall	**0.977(2)**	0.976(2)	0.968(3)	0.969(4)
log-loss	0.130(48)	0.362(48)	0.195(59)	**0.093(14)**

We find from Table 3 that our CASIMAC exhibits a comparatively good overall score on all data sets considered. In particular, its log-loss is better in all cases than that of the GPC. It is also better than the log-loss of all other classifiers except for the wifi data set on which the MLP is superior. Similarly, the accuracy of our approach is also better than that of the other candidates except for the climate data set on which GPC and kNN perform slightly better. Taking the uncertainties of the results into account, it turns out that in most cases the scores fall within the range of a single standard deviation of each other. In particular the log-loss, however, shows the most discrepancies between the classifiers.

**Fig 8:**
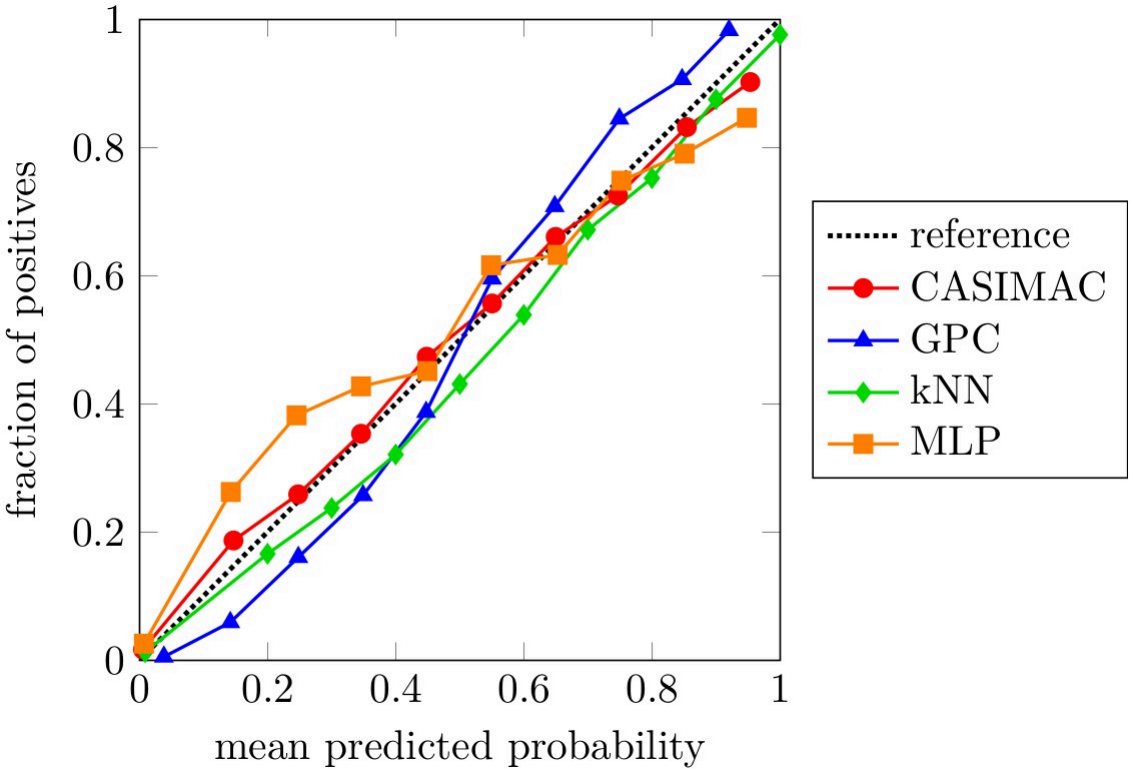
Calibration curves. Calibration curves for the benchmarked classifiers on the pine data set. The closer the curves are to the diagonal reference line, the better the calibration of the respective classifier. In particular, our method exhibits the best calibration properties.

For binary classification problems, it is also interesting to analyze the calibration curves (or reliability diagrams) in addition to the scores [2, 33]. For this purpose, we predict the probability of class 1 for all test samples and discretize the results into ten bins. For each bin, we plot the true fraction of class 1 against the arithmetic mean of the predicted probabilities. For a perfectly calibrated classifier, such a curve corresponds to a diagonal line and deviations from this line can therefore be understood as miscalibrations. As an example, we consider the pine data set and show a typical calibration curve Fig 8.

Clearly, the curve of our CASIMAC is closer to the diagonal reference line than the curves of the other classifiers. In particular, the GPC curve takes on a sigmoidal shape with major deviations from the diagonal at the beginning and the end. In order to obtain a quantitative measure for the observed miscalibrations, we calculate the corresponding area-deviation, that is, the area between each curve and the diagonal reference line. In case of an optimal calibration, this area vanishes, otherwise it is positive. The results are listed in Table 4 and allow us to rank the classifiers by calibration quality: CASIMAC gives by far the best calibration result, followed by kNN and MLP, and with GPC at the very end.

**Table 4:** Calibration score. Calibration score measured in terms of the area-deviation (area between each curve and the diagonal reference line) for the calibration curves from Fig 8, which refer to the pine data set. The best result is highlighted in bold.

Score	CASIMAC	GPC	kNN	MLP
area-deviation	**0.019**	0.062	0.044	0.058

Summarized, our benchmark on different real-world data sets shows that our proposed CASIMAC can compete with other well-established classifiers and exhibits comparably good calibration properties.

### 3.3 Visualization

In order to illustrate the use of our compressed latent space representation for visualization purposes (Section 2.4), we consider a simplified version of the data set alcohol from Table 2 and we refer to this simplified version as alcohol-3. It is obtained from alcohol by merging the last three classes into one and, thus, it has only three instead of five classes (that is, *n* = 3 and *m* = 10).

We train an exemplary CASIMAC using *D* = 25 points from alcohol-3 as training datapoints, while using the remaining *T* = 100 points as test datapoints. As the distance metric *d* underlying the training data transformation *f* of our CASIMAC, we choose the Euclidean distance, and for the hyperparameters *α*, *β*, *k*_*α*_, *k*_*β*_ of *f* we choose

$$\begin{array}{c} \alpha ≔1/2≕\beta \;\text{and}\;k_{\alpha }≔1\;\text{and}\;k_{\beta }≔5. \end{array}$$

As the regression model $\hat{f}$ underlying our CASIMAC, in turn, we choose the same GPR as in our previous benchmarks, see S3 Appendix.

**Fig 9:**
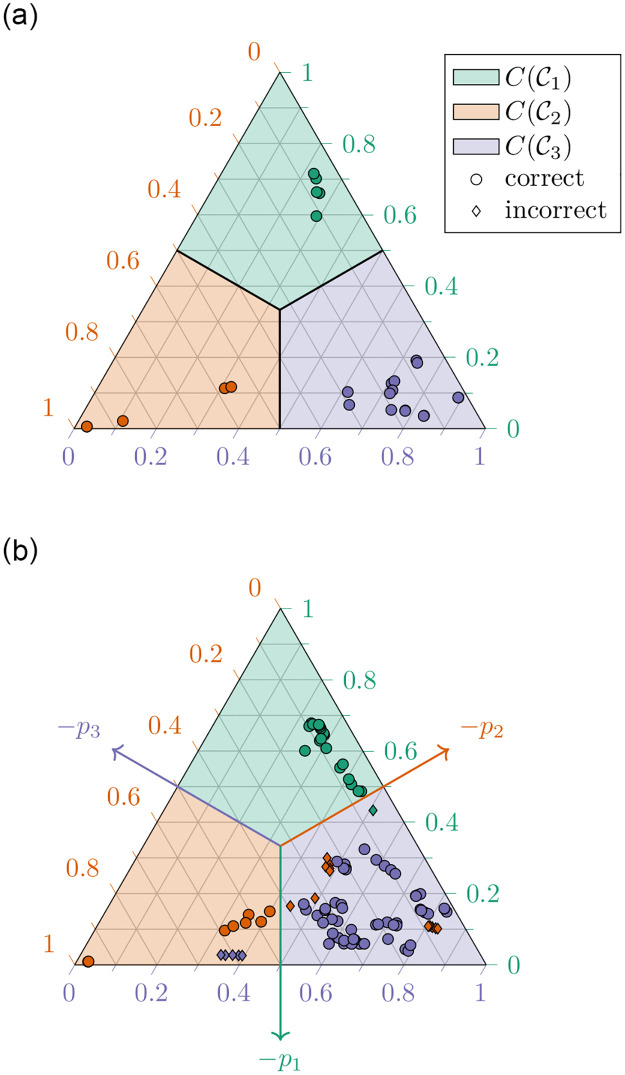
Compressed latent space representation. (a) Compressed latent space representation of the training data. (b) Compressed latent space representation of the test data. Images of (a) the training datapoints under the transformation *C* ∘ *f* and of (b) the test datapoints under the transformation $C\circ \hat{f}$. See (46) and (47), respectively. The color of each point represents its true class, while the form of the markers indicates whether a point is correctly classified (∘) or not (♢) by our CASIMAC. All training datapoints are correctly classified by our construction of *f* and *C*, see (21) and (39). The scales on the three simplex edges indicate the barycentric coordinates of the simplex points (Lemma 14).

In Fig 9, we show the reference simplex S⊂Z with the segmentation induced by the segmentation (12) of Z. So, the differently colored segments in Fig 9 are nothing but the sets $S^{\circ }\cap C_{k}$ and by (39), in turn, these simplex segments are precisely the images of the cone segments $C_{k}$ under the compression map: $S^{\circ }\cap C_{k}=C(C_{k})$ for k∈Y={1,2,3}.

Specifically, Fig 9a displays the compressed latent space representation of the training datapoints *x*_1_, …, *x*_*D*_, that is, the points

$$\begin{array}{c} w_{i}≔C\left(f\left(x_{i}\right)\right)\;\left(i\in \left\{1,\ldots ,D\right\}\right) \end{array}$$ (46)

where *C* is the compression map from (38). In view of (21) and (39), any one of these transformed datapoints *w*_1_, …, *w*_*D*_ belongs to the simplex segment corresponding to its true class label. Also, the distances between points *w*_*i*_ of neighboring classes can indicate inter-class relationships in the original feature space data set. We see from Fig 9a, for instance, that the points *w*_*i*_ belonging to class 1 or 2, respectively, have a larger distance from each other than from the points of class 3. And this leads us to the conclusion that also the original classes 1 and 2 are more clearly separated than are the classes 1 and 3 and, respectively, the classes 2 and 3.

In turn, Fig 9b displays the compressed latent space representation of the test datapoints *x*_*D*+1_, …, *x*_*D*+*T*_, that is, the points

$$\begin{array}{c} w_{i}≔C\left(\hat{f}\left(x_{i}\right)\right)\;\left(i\in \left\{D+1,\ldots ,D+T\right\}\right). \end{array}$$ (47)

Since the regression model is fitted to the training datapoints (*x*_1_, *f*(*x*_1_)), …, (*x*_*D*_, *f*(*x*_*D*_)) only, a test point *w*_*i*_ from (47) can lie in the simplex segment corresponding to its true class label but it can also lie in a simplex segment corresponding to a false class label. In the latter case, our CASIMAC leads to an incorrect class label prediction and the degree of misclassification can be gathered from the degree of misplacement of *w*_*i*_. As we can see from Fig 9b, there are misclassifications between members of class 1 and 3 and especially between members of class 2 and 3, but not between points of the classes 1 and 2, as one would expect from our previous observation about inter-class distances in the training data set. We list the detailed classification results of the test data set in form of a (transposed) confusion matrix in Table 5. As expected, most misclassifications happen between the classes 2 and 3.

**Table 5:** Confusion matrix. Confusion matrix (error matrix): the entry in row *k* and column *l* is the number of test datapoints which belong to class *k* and for which our CASIMAC predicts the class label *l*. Correct classifications (on the diagonal) are highlighted in bold. There are no misclassifications between members of the classes 1 and 2, as can be expected from Fig 9a.

True class	$\hat{y}=1$	$\hat{y}=2$	$\hat{y}=3$
1 (20 members)	**19**	0	1
2 (20 members)	0	**8**	12
3 (60 members)	0	5	**55**

### 3.4 Towards deep learning

Finally, as a proof of concept for a classification task with a larger training data set, we consider the fashion-mnist data set. It consists of 28×28 pixel images of fashion articles in 8-bit grayscale format (i. e., $X≔\{0,\ldots ,255{\}}^{784}$) as described in [34]. In total, there are *D* = 60000 training images and *T* = 10000 test images, which are assigned to *n* = 10 classes. We perform a min-max normalization of the data before we feed it to our classifier, so that the individual features lie within the range [0, 1].

Concerning the training data transformation *f* underlying our CASIMAC, we use two approaches. In the first approach, we make the same ansatz (14) for the training data transformation *f* as in all previous examples, that is,

$$\begin{array}{c} f=f_{\alpha ,\beta ,k_{\alpha },k_{\beta },d}. \end{array}$$ (48)

As the distance metric *d*, we choose the Euclidean distance, and the hyperparameters *α*, *β*, *k*_*α*_, *k*_*β*_ we choose to be

$$\begin{array}{c} \alpha ≔0,\;\beta ≔1,\;k_{\alpha }≔1,\;k_{\beta }≔1. \end{array}$$ (49)

In the second approach, we make a mixture ansatz for the training data transformation, namely, we take *f* to be the average of three training data transformations of the form (14). In short,

$$\begin{array}{c} f=\left(f_{1}+f_{2}+f_{3}\right)/3\;\text{with}\;f_{i}=f_{\alpha ,\beta ,k_{\alpha },k_{\beta },d_{i}} \end{array}$$ (50)

and for all three components *f*_1_, *f*_2_, *f*_3_ we choose the hyperparameters *α*, *β*, *k*_*α*_, *k*_*β*_ as in (49). As the distance metric *d*_1_ for *f*_1_, we again choose the Euclidean distance, but the distance metrics *d*_2_ and *d*_3_ for *f*_2_, *f*_3_ we choose in a problem-specific way, namely as the similarity metrics defined by

$$\begin{array}{c} d_{2}\left(x,x^{\prime }\right)≔1-s_{5}\left(x,x^{\prime }\right)\;\text{and}\;d_{3}\left(x,x^{\prime }\right)≔1-s_{13}\left(x,x^{\prime }\right). \end{array}$$

In these definitions, *s*_*w*_(*x*, *x*′) ∈ [−1, 1] is the structural similarity index for two images *x*, *x*′ with sliding window size *w* [35, 36] and we use the implementation from [37]. In particular, *d*_2_ and *d*_3_ are valid semimetrics because *s*_*w*_ is symmetric and because *s*_*w*_(*x*, *x*′) = 1 if and only if *x* = *x*′, as pointed out on page 106 of [36].

Since our first approach (48) with its purely Euclidean distance metric does not take into account the structural properties of our image data, it can be considered naive. In contrast, our second approach (50) is informed because it brings to bear the fact that the data consists of images, between which a structural relationship can be established. A general overview of such informed machine learning techniques can be found, for instance, in [38].

As the regression model $\hat{f}$ underlying our CASIMAC, we take a fully connected neural network, both in the naive and in the informed approach. The network contains a first hidden layer with 100 neurons and a sigmoid activation function and a second hidden layer with 18 neurons and a linear activation function. The output of the second layer is interpreted as the mean and standard deviation of a normal distribution. We optimize the log-likelihood of this distribution with an Adam approach to determine the best model parameters. In total, there are 80318 trainable parameters. This model is realized with the help of Tensorflow-probability [39]. Since we predict a distribution $\hat{q}(\;\cdot \;|x)$ for each input point *x*, we can calculate class label probabilities according to (35).

In Table 6, we summarize the classification results for our CASIMAC based on the naive training data transformation (48) and our CASIMAC based on the informed training data transformation (50). Specifically, we show the top-1 to top-5 accuracy scores, which are based on the probability prediction of the classifier. It turns out that our informed approach is slightly better or equal to the naive approach for all accuracies. A list of benchmarked accuracies for other classifiers can be found in [34], for instance.

**Table 6:** Accuracy for the fashion-mnist data set. Top-1 to top-5 accuracy of our naive and of our informed CASIMAC on the fashion-mnist data set. In the naive approach we use a purely Euclidean distance metric between the images, whereas the informed approach also takes the structrual image similarity into account. The best scores are highlighted in bold.

Accuracy	naive	informed
top-1	0.874	**0.880**
top-2	**0.961**	**0.961**
top-3	**0.984**	**0.984**
top-4	0.990	**0.993**
top-5	0.993	**0.996**

In contrast to the approach from [7], we use the neural network to directly perform the latent space mapping instead of linking the network to a series of GPs. Additionally, our network directly predicts the estimated mean and variance of the latent space mapping. It would, however, be a promising approach to further improve our results by incorporating a more advanced form of feature extraction like the one from [7].

## 4 Conclusions and outlook

In this paper, we have introduced a novel classifier called CASIMAC for multi-class classification in arbitrary semimetrizable feature spaces. It is based on the idea of transforming the classification problem into a regression problem. We achieve this by mapping the training data onto a latent space with a simplex-like geometry and subsequently fitting a regression model to the transformed training data. With the help of this regression model, the predictions of our classifier for the class labels and for the class label probabilities can be obtained in a conceptually and computationally simple manner. We have described in detail how our proposed method works and have demonstrated that it can be successfully applied to real-world data sets from various application domains. In particular, we see three major benefits of our approach.

First, it is generic and flexible in the sense that the choice of the particular distance semimetric for our training data transformation and the choice of the regression model underlying our classifier are completely arbitrary. In particular, this capability allows for non-numeric features. Moreover, it enables the integration of additional expert knowledge in the chosen distance metric [38]. For instance, to classify molecules a distance measure reflecting stoichiometry and configuration variations could be applied [40]. Another possible strategy would be to infer the distance metric from the data itself, possibly based on certain informed assumptions [41]. Similarly, expert knowledge could be brought to bear in the training of the regression model, for example, in the form of shape constraints [42–44].

Second, the intuitive latent space representation with its simple geometric concept has a direct interpretation. In particular, this can be exploited to visually detect inter-class relationships and is especially useful for classification problems with a large feature space dimension and a small number of classes.

Third, as our benchmarks have shown, our method leads to classifiers with comparably good prediction and calibration qualities. To determine class probabilities, we only require a regression model with probabilistic predictions. Also, the effort of computing these class probability predictions is quite low, especially compared to the computational effort necessary for GPC. In particular, no complicated approximations are required in our approach and, for a binary classification problem and a regression model with a normally distributed probabilistic prediction, there even exists a closed-form expression for the class probability predictions.

A challenge of our method is that its training requires the calculation of nearest datapoint neighbors, which is computationally expensive for larger data sets [45]. It would be a natural starting point for further studies to investigate how this computational limitation in the training of our classifiers can be overcome. A related challenge of our method is that the tuning of hyperparameters can be costly. Instead of using a cross-validated grid search like we did in this paper, it could be advantageous to consider more elaborate strategies, for example, to infer the hyperparameters from the statistical properties of the training data. We leave this topic as an open question for future research.

## Supporting information

S1 Appendix A: Segmentation of latent space and core properties of calibrated simplex-mapping classifiers. Mathematical background of the method including detailed proofs.
(PDF)

S2 Appendix B: Implementation. Description of the Python implementation of the proposed method.
(PDF)

S3 Appendix C: Benchmark. Description of the hyperparameters for the numerical benchmarks.
(PDF)
