# Supplementary material for: Calibrated simplex-mapping classification
Source: PLoS One. 2023 Jan 17;18(1):e0279876. doi: 10.1371/journal.pone.0279876 (PMC9844900; doi:10.1371/journal.pone.0279876)
Supplement: S1 Appendix — Mathematical background of the method including detailed proofs. (PDF) [file pone.0279876.s001.pdf]

A Segmentation of latent space and core properties of calibrated simplex-mapping classifiers

In this appendix, we collect some supplementary results needed for a detailed and mathematically sound understanding of our CASIMAC methodology. In particular, we prove the core properties of our segmentation of the latent space and of our CASIMAC. As in the main text, n always stands for an integer larger than or equal to 2 and

$$\mathcal{Y} := \{1, \ldots, n\} \qquad \text{and} \qquad \mathcal{Z} := \mathbb{R}^{n-1} \tag{51}$$

denote the corresponding set of class labels and, respectively, the corresponding latent space. Also, $\|z\|_2 := (z \cdot z)^{1/2}$ always stands for the standard ℓ^2-norm of $z \in \mathcal{Z}$, which is induced by the standard scalar product $(z, w) \mapsto z \cdot w = \sum_{i=1}^{n-1} z_i w_i$ on $\mathcal{Z}$.

A.1 Simplices

We begin by briefly discussing $(n-1)$-simplices in $\mathcal{Z}$ [1] and, especially, regular $(n-1)$-simplices in $\mathcal{Z}$ [2]. Simplices are the generalization of triangles to arbitrary dimensions: for example, an $(n-1)$-simplex corresponds to a line segment for $n = 2$, a triangle for $n = 3$, and a tetrahedron for $n = 3$. Specifically, a subset $\mathcal{S}$ of $\mathcal{Z}$ is called an $(n-1)$-*dimensional simplex* or, for short, an $(n-1)$-*simplex* in $\mathcal{Z}$ iff it is the convex hull of n affinely independent points $p_1, \ldots, p_n$ in $\mathcal{Z}$, that is, iff

$$\mathcal{S} = \operatorname{conv}\{p_1, \ldots, p_n\} := \left\{ z \in \mathcal{Z} : z = \sum_{i \in \mathcal{Y}} \lambda_i p_i \text{ for some } \lambda_i \in [0, \infty) \text{ with } \sum_{i \in \mathcal{Y}} \lambda_i = 1 \right\}$$

for some affinely independent points $p_1, \ldots, p_n \in \mathcal{Z}$. As usual, affine independence of the points $p_1, \ldots, p_n$ simply means that, for some (and hence every) $k \in \mathcal{Y}$, the set $\{p_i - p_k : i \in \mathcal{Y} \setminus \{k\}\}$ of all vectors connecting p_k with the points p_i is linearly independent. It is straightforward to verify that the affinely independent points $p_1, \ldots, p_n$ with $\mathcal{S} = \operatorname{conv}\{p_1, \ldots, p_n\}$ are uniquely determined by the set $\mathcal{S}$, namely as the extreme points of $\mathcal{S}$. (See [3] (Proposition 1.17), for instance.) These uniquely determined extreme points $p_1, \ldots, p_n$ of $\mathcal{S}$ are also called the *vertices of* $\mathcal{S}$. If $\mathcal{S}$ is an $(n-1)$-simplex with vertices $p_1, \ldots, p_n$, then the point

$$c := \frac{1}{n} \sum_{i \in \mathcal{Y}} p_i \in \operatorname{conv}\{p_1, \ldots, p_n\} = \mathcal{S} \tag{52}$$

is called the *barycenter of* $p_1, \ldots, p_n$ and, by extension, the *barycenter of* $\mathcal{S}$. Also, the simplex $\mathcal{S}$ is called *regular* iff all of its $n(n-1)/2$ edges have the same (positive) length, that is, iff

$$\|p_i - p_j\|_2 = l \qquad (i, j \in \mathcal{Y} \text{ with } i \neq j) \tag{53}$$

for some $l \in (0, \infty)$, which is also called the edge length of $\mathcal{S}$. See page 121 of [2], for instance. In our proofs, we repeatedly use the following straightforward fact.

Lemma 1. *Suppose $p_1, \ldots, p_n$ are the vertices of an $(n-1)$-simplex in $\mathcal{Z}$ with barycenter 0. Then*

$$\operatorname{span}\{p_i : i \in \mathcal{Y} \setminus \{k\}\} = \mathcal{Z} \tag{54}$$

for every $k \in \mathcal{Y}$. In particular, for every $k \in \mathcal{Y}$, the $(n-1)$-element subset $\{p_i : i \in \mathcal{Y} \setminus \{k\}\}$ of the vertex set is linearly independent.

Proof. We give the straightforward proof for the sake of completeness. Since, by the definition of simplex vertices, the set $\{p_i - p_k : i \in \mathcal{Y} \setminus \{k\}\}$ is a linearly independent set of $n-1$ vectors in the $(n-1)$-dimensional vector space $\mathcal{Z}$, we see that

$$\mathcal{Z} = \operatorname{span}\{p_i - p_k : i \in \mathcal{Y} \setminus \{k\}\} \tag{55}$$

for every $k \in \mathcal{Y}$. Since, moreover, the barycenter of the vertices $p_1, \dots, p_n$ is 0, we further see that

$$p_k = -\sum_{i \in \mathcal{Y} \setminus \{k\}} p_i \in \operatorname{span}\{p_i : i \in \mathcal{Y} \setminus \{k\}\} \tag{56}$$

and therefore

$$\operatorname{span}\{p_i - p_k : i \in \mathcal{Y} \setminus \{k\}\} \subset \operatorname{span}\{p_i : i \in \mathcal{Y} \setminus \{k\}\} \subset \mathcal{Z} \tag{57}$$

for every $k \in \mathcal{Y}$. Combining (55) and (57), we obtain the asserted equality (54). In particular, this equality implies the asserted linear independence by a trivial dimensionality argument. ■

We now construct an explicit example of the kind of simplices that underlie our segmentation of $\mathcal{Z}$ and our CASIMAC. In other words, we construct a regular $(n-1)$-simplex in $\mathcal{Z}$ with barycenter 0 and with vertices $p_1, \dots, p_n$ at unit distance from 0, that is,

$$\sum_{i \in \mathcal{Y}} p_i = 0 \qquad \text{and} \qquad \|p_i\|_2 = 1 \qquad (i \in \mathcal{Y}). \tag{58}$$

In the extreme special case $n = 2$, it is clear that there is only one such simplex, namely the line segment between the vertices -1 and 1 in $\mathcal{Z} = \mathbb{R}$, and we usually index these two vertices increasingly (instead of decreasingly) then, that is,

$$p_1 = -1 \qquad \text{and} \qquad p_2 = 1. \tag{59}$$

In the following, we show that in higher dimensions there still is only one such simplex – provided that we identify mutually congruent simplices.

Proposition 2. *A regular $(n-1)$-simplex with vertices $p_1, \dots, p_n$ satisfying (58) exists in $\mathcal{Z}$. And, moreover, all other regular $(n-1)$-simplices in $\mathcal{Z}$ with (58) are congruent to it.*

Proof. We begin by constructing just any regular $(n-1)$-simplex $\operatorname{conv}\{q_1, \dots, q_n\}$ in $\mathcal{Z}$. In order to do so, we make the ansatz

$$q_i = e_i \qquad (i \in \{1, \dots, n-1\}) \qquad \text{and} \qquad q_n = (\alpha_1, \dots, \alpha_{n-1}), \tag{60}$$

where $e_1, \dots, e_{n-1}$ denote the canonical unit vectors in $\mathbb{R}^{n-1}$ and the coordinates α_1, $\dots, \alpha_{n-1} \in \mathbb{R}$ are to be determined such that the vertices $q_1, \dots, q_n$ all have the same distance from each other. Since, for $i, j \in \{1, \dots, n-1\}$,

$$\|q_i - q_j\|_2^2 = \|e_i - e_j\|_2^2 = 2 \qquad (i \neq j) \tag{61}$$

$$\|q_i - q_n\|_2^2 = \|e_i - q_n\|_2^2 = 1 - 2e_i \cdot q_n + \|q_n\|_2^2 = 1 - 2\alpha_i + \sum_{l=1}^{n-1} \alpha_l^2 \tag{62}$$

by virtue of (60), we have to choose the coordinates

$$\alpha_i = \alpha := (\sum_{l=1}^{n-1} \alpha_l^2 - 1)/2 \qquad (i \in \{1, \dots, n-1\}) \tag{63}$$

to be independent of i. Inserting (63) into (62), we get a quadratic equation in α which has the solutions

$$\alpha = \alpha_\pm := (1 \pm \sqrt{n})/(n-1). \tag{64}$$

In view of these preliminary considerations, we define the points $q_1, \dots, q_n \in \mathcal{Z}$ by

$$q_i := e_i \qquad (i \in \{1, \dots, n-1\}) \qquad \text{and} \qquad q_n := \frac{1+\sqrt{n}}{n-1}(1, \dots, 1). \tag{65}$$

It is then clear that $\operatorname{span}\{q_i - q_n : i \in \{1, \dots, n-1\}\} = \mathcal{Z}$ and thus $q_1, \dots, q_n$ are the vertices of an $(n-1)$-simplex in $\mathcal{Z}$. It is also clear by (61) and (62) that these vertices all have the same distance from each other, namely $\sqrt{2}$. Consequently, $q_1, \dots, q_n$ are the vertices of a regular $(n-1)$-simplex in $\mathcal{Z}$. In particular, by shifting them by their barycenter c and by then normalizing, we obtain the vertices

$$p_i := (q_i - c)/\nu \qquad (i \in \mathcal{Y}) \tag{66}$$

of a regular $(n-1)$-simplex satisfying (58), as desired. Specifically,

$$c := \frac{1}{n}\sum_{i \in \mathcal{Y}} q_i = \frac{1 + 1/\sqrt{n}}{n-1}(1, \dots, 1) \qquad \text{and} \qquad \nu := \|q_i - c\|_2 = \sqrt{1 - 1/n}. \tag{67}$$

It remains to prove that any other regular $(n-1)$-simplex satisfying (58) is congruent to the simplex constructed above. In order to do so, we have only to notice that any such other simplex has the same edge length as the simplex constructed above, by [4] (first part of Corollary 3), and that all regular simplices with equal edge length are congruent to each other, again by [4] (Theorem 2). ■

Lemma 3. *Suppose $p_1, \dots, p_n$ are the vertices of a regular $(n-1)$-simplex in $\mathcal{Z}$ with (58). Then*

$$p_i \cdot p_j = 1 \qquad (i = j) \qquad \textit{and} \qquad p_i \cdot p_j = -1/(n-1) \qquad (i \neq j). \tag{68}$$

Proof. An immediate consequence of [4] (second part of Corollary 3) or of [5] (proof of Theorem 1). ■

A.2 Simplex-induced segmentation of the latent space

In this section, we introduce the segmentation of the latent space $\mathcal{Z}$ underlying our classification method and establish the core properties of this segmentation. We choose a segmentation into n convex cone segments $\mathcal{C}_1, \dots, \mathcal{C}_n$ and we define these cone segments in terms of the vertices of an $(n-1)$-simlex with barycenter 0. As usual, a convex cone in $\mathcal{Z}$ is a convex subset of $\mathcal{Z}$ that is closed under addition and under scalar multiplication with positive scalars [3] (Proposition 1.7).

Lemma 4. *Suppose $p_1, \dots, p_n$ are the vertices of an $(n-1)$-simplex in $\mathcal{Z}$ with (58.a) and, for every $k \in \mathcal{Y}$, let*

$$\mathcal{C}_k := \left\{ z \in \mathcal{Z} : z = \sum_{i \in \mathcal{Y}\setminus\{k\}} c_i \cdot (-p_i) \textit{ for some } c_i \in [0, \infty) \right\} \tag{69}$$

be the convex cone generated by the mirrored vertices $-p_i$ for $i \in \mathcal{Y} \setminus \{k\}$. Suppose further that z is a point in $\mathcal{Z}$ having the representation

$$z = \sum_{i \in \mathcal{Y}} r_i \cdot (-p_i) \tag{70}$$

for some $r_1, \ldots, r_n \in \mathbb{R}$. Then, for every $k \in \mathcal{Y}$, one has the following equivalence: $z \in \mathcal{C}_k$ if and only if $r_k \le r_i$ for all $i \in \mathcal{Y}$.

Proof. Choose and fix an arbitrary $k \in \mathcal{Y}$. It then follows from (70) with the help of (56) that

$$z = r_k \sum_{i \in \mathcal{Y} \setminus \{k\}} p_i + \sum_{i \in \mathcal{Y} \setminus \{k\}} r_i \cdot (-p_i) = \sum_{i \in \mathcal{Y} \setminus \{k\}} (r_i - r_k) \cdot (-p_i). \tag{71}$$

If $r_k \le r_i$ for all $i \in \mathcal{Y}$, then it immediately follows from (71) and the definition (69) of $\mathcal{C}_k$ that $z \in \mathcal{C}_k$, as desired. If, conversely, $z \in \mathcal{C}_k$, then

$$z = \sum_{i \in \mathcal{Y} \setminus \{k\}} c_i \cdot (-p_i) \tag{72}$$

for some $c_i \in [0, \infty)$ by the definition (69) of $\mathcal{C}_k$ and, therefore, it follows by virtue of (71) and (72) that

$$\sum_{i \in \mathcal{Y} \setminus \{k\}} (r_i - r_k) \cdot p_i = -z = \sum_{i \in \mathcal{Y} \setminus \{k\}} c_i \cdot p_i. \tag{73}$$

Since $\{p_i : i \in \mathcal{Y} \setminus \{k\}\}$ is linearly independent by Lemma 1, we conclude from (73) that $r_i - r_k = c_i \ge 0$ for all $i \in \mathcal{Y} \setminus \{k\}$ and therefore $r_k \le r_i$ for all $i \in \mathcal{Y}$, as desired. ∎

Lemma 5. *Suppose $p_1, \ldots, p_n$ are the vertices of an $(n-1)$-simplex in $\mathcal{Z}$ with (58.a) and let $\mathcal{C}_k$ be the cone defined in (69). Then the closure $\overline{\mathcal{C}}_k$ and the interior $\mathcal{C}_k^\circ$ of $\mathcal{C}_k$ are given, respectively, by*

$$\overline{\mathcal{C}}_k = \mathcal{C}_k \quad \text{and} \quad \mathcal{C}_k^\circ = \Big\{ z \in \mathcal{Z} : z = \sum_{i \in \mathcal{Y} \setminus \{k\}} c_i \cdot (-p_i) \text{ for some } c_i \in (0, \infty) \Big\}. \tag{74}$$

In particular, $\mathcal{C}_k$ is a closed set in $\mathcal{Z}$.

Proof. Choose and fix an arbitrary $k \in \mathcal{Y}$ and let $\varphi_k : \mathbb{R}^{n-1} \to \mathcal{Z}$ be the linear map defined by $\varphi_k(e_i) = -p_i$ for $i \in \{1, \ldots, k-1\}$ and $\varphi_k(e_i) = -p_{i+1}$ for $i \in \{k, \ldots, n-1\}$, where $e_1, \ldots, e_{n-1}$ denote the canonical unit vectors in $\mathbb{R}^{n-1}$. It is then obvious that the cone $\mathcal{C}_k$ is the image under φ_k of the non-negative orthant $P := [0, \infty)^{n-1}$ in $\mathbb{R}^{n-1}$. In short,

$$\mathcal{C}_k = \varphi_k(P). \tag{75}$$

It is also clear, by the linear independence of $\{\varphi_k(e_i) : i \in \{1, \ldots, n-1\}\}$ (Lemma 1), that φ_k is a linear isomorphism of $\mathcal{Z} = \mathbb{R}^{n-1}$ and thus also a homeomorphism of $\mathcal{Z}$ (Theorem VII.1.6 in of [6]). Since obviously $\overline{P} = P$ and $P^\circ = (0, \infty)^{n-1}$, we see by (75) and [7] (Theorem III.11.3 and Theorem III.11.4) that

$$\overline{\mathcal{C}}_k = \overline{\varphi_k(P)} = \varphi_k(\overline{P}) = \varphi_k(P) = \mathcal{C}_k, \qquad \mathcal{C}_k^\circ = (\varphi_k(P))^\circ = \varphi_k(P^\circ) = \varphi_k((0, \infty)^{n-1}).$$

Clearly, $\varphi_k((0, \infty)^{n-1})$ is equal to the set on the right-hand side of (74.b) and thus the proof is finished. ∎

With the help of the preceding lemmas, we can prove that the cone segments cover the whole latent space and that they are essentially non-overlapping (up to boundary points). It should be noticed that for this result, we do not have to require the underlying $(n-1)$-simplex to be regular.

Proposition 6. *Suppose $p_1, \dots, p_n$ are the vertices of an $(n-1)$-simplex in $\mathcal{Z}$ with (58.a) and let $\mathcal{C}_1, \dots, \mathcal{C}_n$ be the cones defined in* (69)*. Then, for every $k \in \mathcal{Y}$, the vertex p_k lies on the central ray*

$$\left\{ z \in \mathcal{Z} : z = \sum_{i \in \mathcal{Y} \setminus \{k\}} c \cdot (-p_i) \text{ for some } c \in [0, \infty) \right\} \tag{76}$$

of the cone $\mathcal{C}_k$. Also, the cones $\mathcal{C}_1, \dots, \mathcal{C}_n$ cover the whole of $\mathcal{Z}$, and any two cones $\mathcal{C}_k$ and $\mathcal{C}_l$ for $k \neq l$ overlap only at their boundaries. In short,

$$\mathcal{Z} = \bigcup_{k \in \mathcal{Y}} \mathcal{C}_k \qquad \text{and} \qquad \mathcal{C}_k^{\circ} \cap \mathcal{C}_l = \emptyset \qquad (k \neq l). \tag{77}$$

Proof. In view of (56), it is obvious that the vertex p_k lies on the central ray (76) of $\mathcal{C}_k$ for every $k \in \mathcal{Y}$.

In order to prove (77.a), we have only to show that every $z \in \mathcal{Z}$ is contained in some $\mathcal{C}_k$. Let $z \in \mathcal{Z}$ be an arbitrary point in $\mathcal{Z}$. It then follows by (54) that z has a representation of the form (70) for some $r_1, \dots, r_n \in \mathbb{R}$. So, by Lemma 4, we see that $z \in \mathcal{C}_k$ for every k with $r_k = \min_{i \in \mathcal{Y}} r_i$.

In order to prove (77.b), we argue by contradiction. Let $k, l \in \mathcal{Y}$ be fixed with $k \neq l$ and assume, for the sake of argument, that there exists a point $z \in \mathcal{Z}$ with

$$z \in \mathcal{C}_k^{\circ} \cap \mathcal{C}_l. \tag{78}$$

In view of (54), z has a representation of the form (70) for some $r_1, \dots, r_n \in \mathbb{R}$. Since $z \in \mathcal{C}_k \cap \mathcal{C}_l$ by assumption (78), we therefore see by Lemma 4 that

$$r_k = \min_{i \in \mathcal{Y}} r_i = r_l. \tag{79}$$

In order to arrive at a contradiction, we consider suitable perturbations z^{ε} of z, namely

$$z^{\varepsilon} := \sum_{i \in \mathcal{Y}} r_i^{\varepsilon} \cdot (-p_i) \qquad (\varepsilon \in (0, \infty)), \tag{80}$$

where $r_i^{\varepsilon} := r_i + \varepsilon$ for $i \in \mathcal{Y} \setminus \{l\}$ and $r_l^{\varepsilon} := r_l$. It is then clear by (79) that $r_l^{\varepsilon} = r_l = r_k < r_k + \varepsilon = r_k^{\varepsilon}$ for every $\varepsilon \in (0, \infty)$ and therefore we see by Lemma 4 that

$$z^{\varepsilon} \notin \mathcal{C}_k \qquad (\varepsilon \in (0, \infty)). \tag{81}$$

Since, on the other hand, $z \in \mathcal{C}_k^{\circ}$ by assumption (78) and the perturbations z^{ε} obviously converge to z, we also see that

$$z^{\varepsilon} \in \mathcal{C}_k \qquad (\varepsilon \in (0, \varepsilon_0]) \tag{82}$$

for some sufficiently small $\varepsilon_0 > 0$. Contradiction between (81) and (82)! So, our assumption (78) cannot be true, as desired. ∎

If we additionally require the $(n-1)$-simplex from the previous proposition to be regular, we can further prove that the corresponding cone segments are mutually congruent.

Proposition 7. *Suppose $p_1, \dots, p_n$ are the vertices of a regular $(n-1)$-simplex in $\mathcal{Z}$ with* (58) *and let $\mathcal{C}_1, \dots, \mathcal{C}_n$ be the cone segments defined in* (69)*. Then any two of these cone segments are congruent to each other.*

Proof. We proceed in three steps and, for the entire proof, we fix $k, l \in \mathcal{Y}$ with $k \neq l$. As a first step, we show that the mid-perpendicular hyperplane

$$\mathcal{M}_{kl} := \{z \in \mathcal{Z} : z \cdot (p_k - p_l) = 0\} \tag{83}$$

between the vertices p_k and p_l is equal to the subspace of $\mathcal{Z}$ spanned by all other vertices, that is,

$$\mathcal{M}_{kl} = \operatorname{span}\{p_i : i \in \mathcal{Y} \setminus \{k, l\}\}. \tag{84}$$

Indeed, in view of Lemma 3, we have $p_i \cdot (p_k - p_l) = 0$ for all $i \in \mathcal{Y} \setminus \{k, l\}$ and therefore

$$\{p_i : i \in \mathcal{Y} \setminus \{k, l\}\} \subset \mathcal{M}_{kl}. \tag{85}$$

Since $\{p_i : i \in \mathcal{Y} \setminus \{k, l\}\}$ is a linearly independent set of $n-2$ vectors by Lemma 1 and $\mathcal{M}_{kl}$ is an $(n-2)$-dimensional subspace of $\mathcal{Z}$, the asserted equality (84) follows from (85) and a trivial dimensionality argument.

As a second step, we show that the segment $\mathcal{C}_l$ is the mirror image of the segment $\mathcal{C}_k$ under the reflection ρ_{kl} at the mid-perpendicular hyperplane $\mathcal{M}_{kl}$, that is,

$$\rho_{kl}(\mathcal{C}_k) = \mathcal{C}_l, \tag{86}$$

Indeed, the reflection at $\mathcal{M}_{kl}$ is the linear map ρ_{kl} given by

$$\rho_{kl}(z) = \pi_{kl}(z) - (z - \pi_{kl}(z)) = 2\pi_{kl}(z) - z \qquad (z \in \mathcal{Z}), \tag{87}$$

where π_{kl} is the orthogonal projection onto $\mathcal{M}_{kl}$. It then follows by the first step that $\pi_{kl}(p_i) = p_i$ for all $i \in \mathcal{Y} \setminus \{k, l\}$ and that $\pi_{kl}(p_k) = (p_k + p_l)/2 = \pi_{kl}(p_l)$ and therefore

$$\rho_{kl}(p_i) = p_i \quad (i \in \mathcal{Y} \setminus \{k, l\}) \quad \text{and} \quad \rho_{kl}(p_k) = p_l \quad \text{and} \quad \rho_{kl}(p_l) = p_k. \tag{88}$$

And from this, in turn, the asserted equality (86) immediately follows by the definition (69) of the cone segments.

As a third step, we finally establish the asserted congruence of the segments $\mathcal{C}_k$ and $\mathcal{C}_l$. Indeed, this immediately follows by the second step, because reflections obviously are congruence transformations (isometries) of $\mathcal{Z}$. ■

With the preceding results at hand, we can establish a distance-based characterization of our cone segments which is central for an economical computation of our classifier's class label and class label probability predictions $\widehat{y}(x)$ and $\widehat{p}(\,\cdot\,|x)$, respectively. See (108) and (119) below.

Theorem 8. *Suppose $p_1, \dots, p_n$ are the vertices of a regular $(n-1)$-simplex in $\mathcal{Z}$ with* (58) *and let $\mathcal{C}_1, \dots, \mathcal{C}_n$ be the cone segments defined in* (69)*. Then these cone segments can be characterized in terms of the distances from the central vectors $p_1, \dots, p_n$ of the cones $\mathcal{C}_1, \dots, \mathcal{C}_n$. Specifically,*

$$\mathcal{C}_k = \big\{z \in \mathcal{Z} : \|z - p_k\|_2 \le \|z - p_l\|_2 \text{ for all } l \in \mathcal{Y}\big\}. \tag{89}$$

And analogously, for the interiors of the cone segments one has the following characterization:

$$\mathcal{C}_k^\circ = \big\{z \in \mathcal{Z} : \|z - p_k\|_2 < \|z - p_l\|_2 \text{ for all } l \in \mathcal{Y} \setminus \{k\}\big\}. \tag{90}$$

Proof. We proceed in four steps and, for the entire proof, we fix $k \in \mathcal{Y}$. As a first step, we observe that the set on the right-hand side of (89) is nothing but the intersection of the half-spaces

$$\mathcal{H}_{kl}(p_k) := \{z \in \mathcal{Z} : z \cdot (p_k - p_l) \geq 0\} \tag{91}$$

for all $l \in \mathcal{Y} \setminus \{k\}$, that is, the half-spaces confined by the mid-perpendicular hyperplanes $\mathcal{M}_{kl}$ from (83) and stretching to the side of p_k (instead of its mirror image p_l). In other words, as a first step we observe that

$$\big\{z \in \mathcal{Z} : \|z - p_k\|_2 \leq \|z - p_l\|_2 \text{ for all } l \in \mathcal{Y}\big\} = \bigcap_{l \in \mathcal{Y}\setminus\{k\}} \mathcal{H}_{kl}(p_k). \tag{92}$$

Indeed, by (58.b) we immediately obtain

$$\|z - p_k\|_2^2 = \|z\|_2^2 - 2z \cdot p_k + 1 \qquad \text{and} \qquad \|z - p_l\|_2^2 = \|z\|_2^2 - 2z \cdot p_l + 1 \tag{93}$$

for every $l \in \mathcal{Y}$ and every $z \in \mathcal{Z}$. And from this, in turn, the asserted equality (92) readily follows.

As a second step, we show that the segment $\mathcal{C}_k$ is contained in the right-hand side of (89) or, equivalently (by the first step), that

$$\mathcal{C}_k \subset \bigcap_{l \in \mathcal{Y}\setminus\{k\}} \mathcal{H}_{kl}(p_k). \tag{94}$$

So, let $z \in \mathcal{C}_k$. We can then represent z in the form (72) with some $c_i \in [0, \infty)$ and we can thus conclude, using Lemma 3, that

$$z \cdot (p_k - p_l) = c_l(-p_l) \cdot (p_k - p_l) + \sum_{i \in \mathcal{Y}\setminus\{k,l\}} c_i(-p_i) \cdot (p_k - p_l) = c_l(1 + 1/(n-1)) \geq 0$$

for every $l \in \mathcal{Y} \setminus \{k\}$. And therefore, z belongs to the set on the right-hand side of (94), as desired.

As a third step, we show that conversely the right-hand side of (89) is contained in the segment $\mathcal{C}_k$ or, equivalently (by the first step), that

$$\bigcap_{l \in \mathcal{Y}\setminus\{k\}} \mathcal{H}_{kl}(p_k) \subset \mathcal{C}_k. \tag{95}$$

So, let $z \in \bigcap_{l \in \mathcal{Y}\setminus\{k\}} \mathcal{H}_{kl}(p_k)$. We then have $z \cdot (p_k - p_l) \geq 0$ for every $l \in \mathcal{Y} \setminus \{k\}$ and therefore we see, using Lemma 3, that the perturbations

$$z^\varepsilon := z + \varepsilon w := z + \varepsilon \sum_{i \in \mathcal{Y}\setminus\{k\}} (p_k - p_i) \qquad (\varepsilon \in (0, \infty)) \tag{96}$$

of z satisfy the strict inequality

$$z^\varepsilon \cdot (p_k - p_l) = z \cdot (p_k - p_l) + \varepsilon \|p_k - p_l\|_2^2 + \varepsilon(n-2)(1 + 1/(n-1)) > 0 \tag{97}$$

for every $l \in \mathcal{Y} \setminus \{k\}$ and every $\varepsilon \in (0, \infty)$. So, by (93) with z replaced by z^ε, this strict inequality (97) implies

$$\|z^\varepsilon - p_k\|_2^2 < \|z^\varepsilon - p_l\|_2^2 \qquad (l \in \mathcal{Y} \setminus \{k\} \text{ and } \varepsilon \in (0, \infty)). \tag{98}$$

And from (98), in turn, we can easily conclude that

$$z^\varepsilon \in \mathcal{C}_k \qquad (\varepsilon \in (0, \infty)). \tag{99}$$

Indeed, by (77.a), we see that for every $\varepsilon \in (0, \infty)$ there exists an index $k' = k'_\varepsilon \in \mathcal{Y}$ such that $z^\varepsilon \in \mathcal{C}_{k'}$ and therefore, by the first and the second step with k replaced by k',

$$\|z^\varepsilon - p_{k'}\|_2 \leq \|z^\varepsilon - p_l\|_2 \qquad (l \in \mathcal{Y} \setminus \{k'\}). \tag{100}$$

Clearly, this is compatible with (98) only if $k' = k$. Consequently, we must have $k' = k$ so that $z^\varepsilon \in \mathcal{C}_{k'} = \mathcal{C}_k$ and (99) is established. Since the perturbations z^ε obviously converge to z, we see from (99) and (74.a) that $z \in \mathcal{C}_k$, as desired.

As a fourth step, we finally establish the asserted equalities (89) and (90). Indeed, (89) is an immediate consequence of (92), (94) and (95). And (90), in turn, is readily obtained by noting that the interior of the half-space $\mathcal{H}_{kl}(p_k)$ is given by

$$(\mathcal{H}_{kl}(p_k))^\circ = \{z \in \mathcal{Z} : z \cdot (p_k - p_l) > 0\} \tag{101}$$

and by then taking the interior on both sides of the inclusions (94) and (95). ■

In view of the normalization condition (58.b) on the vertices of our simplex, the norm characterization (89) immediately translates to a scalar-product characterization of our cone segments. We will use it to prove the invariance of the cone segments under the compression and inflation maps from (130) and (131) below.

Corollary 9. *Suppose $p_1, \dots, p_n$ are the vertices of a regular $(n-1)$-simplex in $\mathcal{Z}$ with* (58) *and let $\mathcal{C}_1, \dots, \mathcal{C}_n$ be the cone segments defined in* (69)*. Then*

$$\mathcal{C}_k = \big\{z \in \mathcal{Z} : z \cdot p_k \geq z \cdot p_l \text{ for all } l \in \mathcal{Y}\big\}. \tag{102}$$

Proof. An immediate consequence of (89) in conjunction with (93). ■

A.3 Core properties of calibrated simplex-mapping classifiers

In this section, we establish the core properties of our CASIMAC. We begin by proving that the underlying training data transformation f maps every training datapoint x_i to the interior $\mathcal{C}^\circ_{y(x_i)}$ of the cone segment corresponding to the true class label $y(x_i) = y_i$.

Proposition 10. *Suppose $p_1, \dots, p_n$ are the vertices of a regular $(n-1)$-simplex in $\mathcal{Z}$ with* (58) *and let $\mathcal{C}_1, \dots, \mathcal{C}_n$ be the cone segments defined in* (69)*. Suppose further that $\mathcal{D} = \{(x_i, y_i) : i \in \{1, \dots, D\}\}$ is a finite subset of $\mathcal{X} \times \mathcal{Y}$ and that the map $f : \mathcal{D}_\mathcal{X} := \{x_1, \dots, x_D\} \to \mathcal{Z}$ is defined as in* (14), (15), *and* (16) *with a semimetric d on $\mathcal{X}$ and hyperparameters $\alpha, \beta \in [0, \infty)$ and $k_\alpha, k_\beta \in \mathbb{N}$ satisfying* (18)*. Then*

$$0 < A_{k_\alpha,d}(x), R_{k_{\beta,d}}(x, y) < \infty \tag{103}$$

for every $x \in \mathcal{D}_\mathcal{X}$ and $y \in \mathcal{Y} \setminus \{y(x)\}$. In particular, $f(x) \in \mathcal{C}^\circ_{y(x)}$ for every $x \in \mathcal{D}_\mathcal{X}$.

Proof. Choose and fix $x \in \mathcal{D}_\mathcal{X}$. Also, for every $y \in \mathcal{Y}$, let $\mathcal{D}_{\mathcal{X},y} := \{x' \in \mathcal{D}_\mathcal{X} : y(x') = y\}$ be the set of training datapoints belonging to class y and let $c := \min\{|\mathcal{D}_{\mathcal{X},y}| : y \in \mathcal{Y}\}$ be the size of the smallest class in the training data. In order to prove (103), we also fix $y \in \mathcal{Y} \setminus \{y(x)\}$. It is then obvious that

$$|\mathcal{D}_{\mathcal{X},y(x)} \setminus \{x\}| = |\mathcal{D}_{\mathcal{X},y(x)}| - 1 \geq c - 1 \qquad \text{and} \qquad |\mathcal{D}_{\mathcal{X},y} \setminus \{x\}| = |\mathcal{D}_{\mathcal{X},y}| \geq c. \tag{104}$$

Combining this with (18.b) and (18.c), we further see that there exists a subset $X'_\alpha \subset \mathcal{D}_{\mathcal{X},y(x)} \setminus \{x\}$ with $|X'_\alpha| = k_\alpha$ and a subset $X'_\beta \subset \mathcal{D}_{\mathcal{X},y} \setminus \{x\}$ with $|X'_\beta| = k_\beta$ and therefore, by the definition (16) of nearest neighbors,

$$\mathrm{NN}_{k_\alpha,d}(x, \mathcal{D}_{\mathcal{X},y(x)}) < \infty \qquad \text{and} \qquad \mathrm{NN}_{k_\beta,d}(x, \mathcal{D}_{\mathcal{X},y}) < \infty. \tag{105}$$

Since moreover d is a semimetric, we have $\sum_{x' \in X'} d(x,x') > 0$ for every non-empty subset X' of $\mathcal{X} \setminus \{x\}$ and therefore the definition (16) of nearest neighbors further shows that

$$\mathrm{NN}_{k_\alpha,d}(x, \mathcal{D}_{\mathcal{X},y(x)}) > 0 \qquad \text{and} \qquad \mathrm{NN}_{k_\beta,d}(x, \mathcal{D}_{\mathcal{X},y}) > 0. \tag{106}$$

In view of (15), (105) and (106), the assertion (103) is clear. And since

$$f(x) = \sum_{y \in \mathcal{Y} \setminus \{y(x)\}} \Big(\alpha A_{k_\alpha,d}(x) + \beta R_{k_\beta,d}(x,y) \Big) \cdot (-p_y)$$

by (58.a), we finally conclude from (18.a) and (103) in conjunction with (74.b) that $f(x) \in \mathcal{C}^\circ_{y(x)}$, as desired. ■

As an immediate consequence of the norm characterization (89) of our cone segments, we obtain the alternative representation (108) of our classifier's class label predictions $\widehat{y}(x)$, which is much simpler computationally than the geometrically inspired definition (107). Additionally, we prove that for the training datapoints x_i, our classifier correctly predicts the true class label $y(x_i) = y_i$ provided that the regression model perfectly predicts its training datapoints $(x_i, f(x_i)) \in \mathcal{D}^f$.

Corollary 11. *Suppose the assumptions of the previous proposition are satisfied. Suppose further that $\widehat{f} : \mathcal{X} \to \mathcal{Z}$ is an arbitrary map and let $\widehat{y} : \mathcal{X} \to \mathcal{Y}$ be the map defined by*

$$\widehat{y}(x) := \widehat{g}(\widehat{f}(x)) := \min\{y \in \mathcal{Y} : \widehat{f}(x) \in \mathcal{C}_y\} \qquad (x \in \mathcal{X}), \tag{107}$$

where $\widehat{g}(z) := \min\{y \in \mathcal{Y} : z \in \mathcal{C}_y\}$. Then

$$\widehat{y}(x) = \min\Big\{ y \in \mathcal{Y} : \big\|\widehat{f}(x) - p_y\big\|_2 = \min_{l \in \mathcal{Y}} \big\|\widehat{f}(x) - p_l\big\|_2 \Big\} \qquad (x \in \mathcal{X}). \tag{108}$$

If, in addition, $\widehat{f}(x_i) = f(x_i)$ for all $i \in \{1, \dots, D\}$, then

$$\widehat{y}(x_i) = y(x_i) = y_i \qquad (i \in \{1, \dots, D\}). \tag{109}$$

Proof. In view of the norm characterization (89) of our cone segments, the assertion (108) is obvious. If $\widehat{f}(x_i) = f(x_i)$ for all i, then $\widehat{f}(x_i) = f(x_i) \in \mathcal{C}^\circ_{y(x_i)}$ by Proposition 10. Since $\mathcal{C}^\circ_{y(x_i)}$ by (77.b) does not overlap with any other cone segment except for $\mathcal{C}_{y(x_i)}$ itself, we also obtain the assertion (109). ■

As a consequence of the essential disjointness (77.b) of our cone segments, we obtain the convenient alternative representation (111) of our classifier's class label probability predictions $\widehat{p}(\,\cdot\,|x)$.

Corollary 12. *Suppose $p_1, \dots, p_n$ are the vertices of a regular $(n-1)$-simplex in $\mathcal{Z}$ with (58) and let $\mathcal{C}_1, \dots, \mathcal{C}_n$ be the cone segments defined in (69). Suppose further that $\widehat{q}(\,\cdot\,|x)$ for every $x \in \mathcal{X}$ is a probability density on $\mathcal{Z}$ and let*

$$\widehat{p}(y|x) := \int_{\{\widehat{g}=y\}} \widehat{q}(z|x)\,\mathrm{d}z \qquad (x \in \mathcal{X} \text{ and } y \in \mathcal{Y}), \tag{110}$$

where $\widehat{g}(z) := \min\{y \in \mathcal{Y} : z \in \mathcal{C}_y\}$. Then

$$\widehat{p}(y|x) = \int_{\mathcal{C}_y} \widehat{q}(z|x)\,\mathrm{d}z = \int_{\mathcal{C}^\circ_y} \widehat{q}(z|x)\,\mathrm{d}z \qquad (x \in \mathcal{X} \text{ and } y \in \mathcal{Y}). \tag{111}$$

Proof. As a first step, we observe that

$$\mathcal{C}_y^\circ \subset \{z \in \mathcal{Z} : \widehat{g}(z) = y\} \subset \mathcal{C}_y \tag{112}$$

for every $y \in \mathcal{Y}$. Indeed, the second inclusion is just a trivial consequence of the definition of $\widehat{g}$ and the first inclusion directly follows from the fact that $\mathcal{C}_y^\circ$ by (77.b) does not overlap with any cone segment except $\mathcal{C}_y$ itself. As a second step, we show that

$$\partial\mathcal{C}_y = \overline{\mathcal{C}}_y \setminus \mathcal{C}_y^\circ = \mathcal{C}_y \setminus \mathcal{C}_y^\circ \subset \bigcup_{k \in \mathcal{Y}\setminus\{y\}} \mathcal{M}_{yk} \tag{113}$$

for every $y \in \mathcal{Y}$, where $\mathcal{M}_{yk}$ as before is the mid-perpendicular hyperplane between the vertices p_y and p_k. In order to see this, let $y \in \mathcal{Y}$ be fixed and let $z \in \partial\mathcal{C}_y = \mathcal{C}_y \setminus \mathcal{C}_y^\circ$. We then have, on the one hand, that

$$\|z - p_y\|_2 = \min_{l \in \mathcal{Y}} \|z - p_l\|_2 \tag{114}$$

by (89) and, on the other hand, this minimum must be attained also at another index $k \neq y$, that is, there must be a $k \in \mathcal{Y} \setminus \{y\}$ with

$$\|z - p_k\|_2 = \min_{l \in \mathcal{Y}} \|z - p_l\|_2 \,. \tag{115}$$

(If the minimum was attained only for the index y, this would mean that $\|z - p_y\|_2 < \|z - p_l\|_2$ for all $l \neq y$ and therefore we would have $z \in \mathcal{C}_y^\circ$ by (90). Contradiction to our choice of z!) Combining (114) and (115), we see that

$$\|z - p_y\|_2 = \|z - p_k\|_2 \tag{116}$$

for some $k \in \mathcal{Y} \setminus \{y\}$. So, by (83) and (93), we see that z must lie on the mid-perpendicular hyperplane $\mathcal{M}_{yk}$. And thus (113) is proven. Since hyperplanes are null sets w.r.t. Lebesgue measure on $\mathcal{Z}$, we further conclude from (113) that the boundary $\partial\mathcal{C}_y$ of every cone segment is a null set as well. Combining this, in turn, with (112), we immediately obtain the alternative representations of $\widehat{p}(y|x)$ from (111). ∎

In the following, we turn to the computation of our class label probability predictions (110), using standard Monte Carlo approximation techniques. In principle, the following approximation result is true for completely arbitrary probability density functions $\widehat{q}(\,\cdot\,|x)$ on $\mathcal{Z}$, but it does require to draw samples from $\widehat{q}(\,\cdot\,|x)$. In the special case of normal-distribution densities $\widehat{q}(\,\cdot\,|x)$, drawing samples is standard, of course (Section 3.24 of [8], for instance).

Proposition 13. *Suppose $p_1, \dots, p_n$ are the vertices of a regular $(n-1)$-simplex in $\mathcal{Z}$ with (58) and let $\mathcal{C}_1, \dots, \mathcal{C}_n$ be the cone segments defined in (69). Suppose further that $\widehat{q}(\,\cdot\,|x)$ for every $x \in \mathcal{X}$ is a probability density on $\mathcal{Z}$ and let*

$$\widehat{p}(y|x) := \int_{\mathcal{C}_y} \widehat{q}(z|x)\,\mathrm{d}z = \int_{\mathcal{Z}} I_{\mathcal{C}_y}(z)\widehat{q}(z|x)\,\mathrm{d}z \qquad (x \in \mathcal{X} \text{ and } y \in \mathcal{Y}), \tag{117}$$

where $I_{\mathcal{C}_y}$ is the indicator function of the cone segment $\mathcal{C}_y$ in analogy to (36). In the special case where $n = 2$ and where $\widehat{q}(\,\cdot\,|x)$ is normally distributed with mean $\widehat{\mu}(x)$ and variance $\widehat{\sigma}(x)^2$, the class probability predictions $\widehat{p}(y|x)$ have the closed-form representation

$$\widehat{p}(1|x) = \frac{1}{2}\Big(1 - \operatorname{erf}\big(\widehat{\mu}(x)/(\sqrt{2}\,\widehat{\sigma}(x))\big)\Big) = 1 - \widehat{p}(2|x), \tag{118}$$

where the convention (59) *was used. In the general case, the class probability prediction* $\widehat{p}(y|x)$*, for every fixed* $x \in \mathcal{X}$ *and* $y \in \mathcal{Y}$*, can be approximated with probability* 1 *by the sample means*

$$\widehat{p}_N(y|x) := \frac{1}{N}\sum_{i=1}^{N} I_{\mathcal{C}_y}(z_i), \tag{119}$$

where $z_1, \dots, z_N$ *are sampled independently from the probability density* $\widehat{q}(\,\cdot\,|x)$ *and where the binary values* $I_{\mathcal{C}_y}(z_i)$ *are determined by means of the norm characterization* (89) *of the cone segment* $\mathcal{C}_y$*. Additionally, for every fixed sample size* $N \in \mathbb{N}$ *and every* $\varepsilon > 0$*, the probability for* $\widehat{p}_N(y|x)$ *to miss* $\widehat{p}(y|x)$ *by more than* ε *is bounded above by* $1/(4\varepsilon^2 N)$*.*

Proof. In the mentioned special case, we have $\mathcal{C}_1 = (-\infty, 0]$ and $\mathcal{C}_2 = [0, \infty)$ by our convention (59) and, with this, the asserted closed-form expression immediately follows. We therefore move on to discuss the general case now. We point out that all the arguments to come are fairly standard and we give them just for the reader's convenience. So, let $x \in \mathcal{X}$ and $y \in \mathcal{Y}$ be fixed, write $Q = Q_x$ for the probability measure on $\mathcal{Z}$ with density $\widehat{q}(\,\cdot\,|x)$, and let $z_1, \dots, z_N$ be independent random variables with distribution Q. Writing

$$\widehat{p} := \widehat{p}(y|x) \qquad \text{and} \qquad h := h_y := I_{\mathcal{C}_y} \qquad \text{and} \qquad \widehat{p}_N := \widehat{p}_N(y|x) := \frac{1}{N}\sum_{i=1} h \circ z_i, \tag{120}$$

we can express the expectation value of $h \circ z_i$ w.r.t. Q as

$$\mathrm{E}(h \circ z_i) = \int_{\mathcal{Z}} h(z)\widehat{q}(z|x)\,\mathrm{d}z = \widehat{p} \tag{121}$$

for every i and, since $h^2 = h$, we see that the variance of $h \circ z_i$ w.r.t. Q is given by

$$\mathrm{Var}(h \circ z_i) = \mathrm{E}((h \circ z_i)^2) - (\mathrm{E}(h \circ z_i))^2 = \mathrm{E}(h \circ z_i) - (\mathrm{E}(h \circ z_i))^2 = \widehat{p}(1 - \widehat{p}) \tag{122}$$

for every i. Since the z_i are independent and h is bounded, the random variables $h \circ z_i$ are independent and (square) integrable and therefore the strong law of large numbers in conjunction with (121) shows that, as $N \to \infty$, the sample means $\widehat{p}_N$ converge to $\widehat{p}$ with Q-probability 1 (or, put differently, Q-almost surely). Also, Bienaymé's identity in conjunction with (122) shows that

$$\mathrm{Var}(\widehat{p}_N) = \frac{1}{N^2}\sum_{i=1}^{N} \mathrm{Var}(h \circ z_i) = \frac{\widehat{p}(1 - \widehat{p})}{N} \tag{123}$$

for every fixed $N \in \mathbb{N}$. So, by Chebyshev's inequality, we get the upper bound

$$Q(\{|\widehat{p}_N - \widehat{p}| > \varepsilon\}) \le \frac{1}{\varepsilon^2}\int |\widehat{p}_N - \widehat{p}|^2\,\mathrm{d}Q = \frac{\mathrm{Var}(\widehat{p}_N)}{\varepsilon^2} = \frac{\widehat{p}(1 - \widehat{p})}{\varepsilon^2 N} \tag{124}$$

on the Q-probability for $\widehat{p}_N(y|x)$ to miss $\widehat{p}(y|x)$ by more than ε. Since $\widehat{p}$ by definition lies between 0 and 1, the numerator on the right-hand side of (124) can be trivially estimated as $\widehat{p}(1 - \widehat{p}) \le 1/4$. Inserting this estimate into (124), we obtain

$$Q(\{|\widehat{p}_N - \widehat{p}| > \varepsilon\}) \le \frac{1}{4\varepsilon^2 N} \tag{125}$$

for every $N \in \mathbb{N}$ and $\varepsilon > 0$, which is precisely the asserted probabilistic bound on the approximation error. ■

In addition to the bound (125), one could also apply the central limit theorem to get approximate 95%-confidence intervals in terms of the sample means (119) and the (biased) sample variances

$$\widehat{\sigma}_N(y|x)^2 := \frac{1}{N}\sum_{i=1}^{N}\big(h\circ z_i - \widehat{p}_N(y|x)\big)^2 = \widehat{p}_N(y|x)\big(1-\widehat{p}_N(y|x)\big)$$

for sufficiently large sample sizes N. See [9] (Section 5.1), for instance. It should be noticed, however, that it is not so straightforward to determine what actually is a sufficiently large sample size N. A detailed discussion of this topic can be found [8] (Chapter 2).

A.4 Compressing the latent space to a reference simplex

In this section, we show how the latent space can be compressed to a reference simplex in a diffeomorphic and cone-segment-preserving manner. In order to do so, we need a lemma on barycentric coordinates.

Lemma 14. *Suppose $p_1,\dots,p_n$ are the vertices of an $(n-1)$-simplex $\mathcal{S}$ in $\mathcal{Z}$. Then for every point $z\in\mathcal{S}$ there exist unique numbers $\lambda_i=\lambda_i(z)\in[0,\infty)$ for $i\in\mathcal{Y}$, the so-called barycentric coordinates of z w.r.t. $\mathcal{S}$, such that*

$$z=\sum_{i\in\mathcal{Y}}\lambda_i\cdot p_i \qquad \text{and} \qquad \sum_{i\in\mathcal{Y}}\lambda_i = 1 \tag{126}$$

Also, a point $z\in\mathcal{S}$ belongs to the interior $\mathcal{S}^\circ$ of $\mathcal{S}$ if and only if all its barycentric coordinates $\lambda_1(z),\dots,\lambda_n(z)$ w.r.t. $\mathcal{S}$ are strictly positive. And finally, the barycentric-coordinate map $\mathcal{S}\ni z\mapsto(\lambda_1(z),\dots,\lambda_n(z))$ is infinitely differentiable.

Proof. Consider the map $\varphi:\mathbb{R}^{n-1}\to\mathcal{Z}$ defined by $\varphi(\lambda):=\underline{\varphi}(\lambda)+p_n$ for all $\lambda\in\mathbb{R}^{n-1}$, where $\underline{\varphi}$ is the linear map with $\underline{\varphi}(e_i)=p_i-p_n$ for all $i\in\{1,\dots,n-1\}$ and $e_1,\dots,e_{n-1}$ denote the canonical unit vectors in $\mathbb{R}^{n-1}$. It is then clear that

$$\varphi(\lambda)=\sum_{i=1}^{n-1}\lambda_i\underline{\varphi}(e_i)+p_n=\sum_{i=1}^{n-1}\lambda_i\cdot p_i+\left(1-\sum_{i=1}^{n-1}\lambda_i\right)\cdot p_n \tag{127}$$

for every $\lambda=\sum_{i=1}^{n-1}\lambda_i e_i\in\mathbb{R}^{n-1}$ and from this, in turn, it immediately follows that $\mathcal{S}=\operatorname{conv}\{p_1,\dots,p_n\}$ is the image under φ of the lower-left corner $\Delta:=\{\lambda\in[0,\infty)^{n-1}:\sum_{i=1}^{n-1}\lambda_i\le 1\}$ of the unit cube in $\mathbb{R}^{n-1}$. In short,

$$\mathcal{S}=\varphi(\Delta). \tag{128}$$

It also immediately follows from the linear independence of $\{\underline{\varphi}(e_i):i\in\{1,\dots,n-1\}\}$ that $\underline{\varphi}$ is a linear isomorphism of $\mathcal{Z}=\mathbb{R}^{n-1}$ and thus also a diffeomorphism of $\mathcal{Z}$ (Theorem VII.1.6 in conjunction with Example VII.2.3 (a) and Exercise VII.5.1 of [6]). Consequently, the translate φ of $\underline{\varphi}$ is a diffeomorphism of $\mathcal{Z}$ as well. With these preliminary observations, the assertions of the lemma immediately follow. Indeed, by (127) and the bijectivity of φ, the barycentric coordinates of any given $z\in\mathcal{S}$ are uniquely determined by z, namely

$$(\lambda_1(z),\dots,\lambda_{n-1}(z))=\varphi^{-1}(z) \qquad \text{and} \qquad \lambda_n(z)=1-\sum_{i=1}^{n-1}\lambda_i(z) \tag{129}$$

for every $z \in \mathcal{S}$. In view of (129) and the infinite differentiability of φ^{-1}, in turn, the asserted infinite differentiability of $\mathcal{S} \ni z \mapsto (\lambda_1(z), \ldots, \lambda_n(z))$ follows. And finally, since the interior of Δ is obviously given by $\Delta^\circ = \{\lambda \in (0,\infty)^{n-1} : \sum_{i=1}^{n-1} \lambda_i < 1\}$, we see by (128) and [7] (Theorem III.11.3) and (127) that

$$\mathcal{S}^\circ = (\varphi(\Delta))^\circ = \varphi(\Delta^\circ) = \big\{z \in \mathcal{Z} : (126) \text{ holds true with } \lambda_1, \ldots, \lambda_n \in (0,\infty)\big\}$$

which, in turn, yields the asserted characterization of the interior points of $\mathcal{S}$. ■

Proposition 15. *Suppose $p_1, \ldots, p_n$ are the vertices of a regular $(n-1)$-simplex in $\mathcal{Z}$ with (58) and let $\mathcal{C}_1, \ldots, \mathcal{C}_n$ be the cone segments defined in (69). Then the compression map $C : \mathcal{Z} \to \mathcal{S}^\circ$, defined by*

$$C(z) := \sum_{i \in \mathcal{Y}} \mu_i(z) \cdot p_i \quad \textit{with} \quad \mu_i(z) := \exp(\tau p_i \cdot z) / \Big(\sum_{j \in \mathcal{Y}} \exp(\tau p_j \cdot z)\Big) \tag{130}$$

and a fixed $\tau \in (0,\infty)$, is a diffeomorphism of $\mathcal{Z}$ onto $\mathcal{S}^\circ$. Its inverse is given by the inflation map $I : \mathcal{S}^\circ \to \mathcal{Z}$, defined by

$$I(w) := \frac{n-1}{\tau n} \sum_{i \in \mathcal{Y}} \ln(\lambda_i(w)) \cdot p_i \tag{131}$$

with $\lambda_1(w), \ldots, \lambda_n(w)$ being the barycentric coordinates of w w.r.t. $\mathcal{S}$ and τ is the same positive number as in (130). Additionally, C and I leave the segments $\mathcal{C}_1, \ldots, \mathcal{C}_n$ invariant.

Proof. It is clear by the definitions (130) and (131) and by Lemma 14 that the compression C is an infinitely differentiable map from $\mathcal{Z}$ into $\mathcal{S}^\circ$ and that, conversely, the inflation I is an infinitely differentiable map from $\mathcal{S}^\circ$ into $\mathcal{Z}$. We now show that I is the inverse of C or, equivalently, that

$$C(I(w)) = w \qquad (w \in \mathcal{S}^\circ) \qquad \text{and} \qquad I(C(z)) = z \qquad (z \in \mathcal{Z}). \tag{132}$$

In order to verify (132.a), we confirm with the help of Lemma 3 that

$$\tau p_i \cdot I(w) = \ln \lambda_i(w) - \frac{1}{n} \sum_{j \in \mathcal{Y}} \ln \lambda_j(w) \qquad (w \in \mathcal{S}^\circ)$$

and from this, in turn, we arrive at (132.a) in a straightforward manner. In order to verify (132.b), we first notice that the positive numbers $\mu_1(z), \ldots, \mu_n(z)$ from (130) are nothing but the barycentric coordinates $\lambda_1(C(z)), \ldots, \lambda_n(C(z))$ of the point $C(z) \in \mathcal{S}^\circ$ w.r.t. $\mathcal{S}$ and, thus,

$$I(C(z)) = \frac{n-1}{\tau n} \sum_{i \in \mathcal{Y}} \ln(\mu_i(z)) \cdot p_i = \frac{n-1}{n} \sum_{i \in \mathcal{Y}} (p_i \cdot z)\, p_i \qquad (z \in \mathcal{Z}), \tag{133}$$

where for the second equality we used (58.a). With the help of Lemma 3 and again (58.a), we further see that $\frac{n-1}{n} \sum_{i \in \mathcal{Y}} (p_i \cdot p_j)\, p_i = p_j$ for every $j \in \mathcal{Y}$ and, by linearly extending this identity to arbitrary $z = \sum_{j \in \mathcal{Y}} r_j p_j$ (Lemma 1), we conclude that the right-hand side of (133) equals z, as desired. So, (132) is proved, that is, $I = C^{-1}$ is the inverse of C. In particular, by the infinite differentiability of C and I pointed out above, C is a diffeomorphism from $\mathcal{Z}$ onto $\mathcal{S}^\circ$. It thus only remains to prove that C and I leave the segments $\mathcal{C}_1, \ldots, \mathcal{C}_n$ invariant or, in other words, that

$$C(\mathcal{C}_k) = \mathcal{C}_k \cap \mathcal{S}^\circ \qquad \text{and} \qquad I(\mathcal{C}_k \cap \mathcal{S}^\circ) = \mathcal{C}_k \tag{134}$$

for every $k \in \mathcal{Y}$. In order to see this, we notice that

$$C(z) = \sum_{i \in \mathcal{Y}} \mu_i(z) \cdot p_i = \sum_{i \in \mathcal{Y}} (-\mu_i(z)) \cdot (-p_i)$$

and thus, by Lemma 4 and (130) and Corollary 9, the following chain of equivalences holds true for every $k \in \mathcal{Y}$ and $z \in \mathcal{Z}$:

$$C(z) \in \mathcal{C}_k \quad \text{iff} \quad -\mu_k(z) = \min_{l \in \mathcal{Y}} (-\mu_l(z)) \quad \text{iff} \quad p_k \cdot z = \max_{l \in \mathcal{Y}} p_l \cdot z \quad \text{iff} \quad z \in \mathcal{C}_k.$$

In short, $C(z) \in \mathcal{C}_k$ if and only if $z \in \mathcal{C}_k$. And this, in turn, immediately implies the asserted invariance statements (134). ■

References

1. Rockafellar RT. Convex analysis. Princeton Mathematical Series. Princeton, N. J.: Princeton University Press; 1970.
2. Coxeter HSM. Regular polytopes. Dover books on advanced mathematics. Dover, New York; 1973.
3. Tuy H. Convex analysis and global optimization. 2nd ed. Springer; 2016.
4. Schmid J, Heese R, Bortz M. An elementary geometric derivation of the central angle of a regular n-simplex. submitted 2022.
5. Parks HR, Wills DC. An Elementary Calculation of the Dihedral Angle of the Regular n-Simplex. The American Mathematical Monthly. 2002;109(8):756–758.
6. Amann H, Escher J. Analysis II. Birkhäuser; 2008.
7. Dugundji J. Topology. Allyn and Bacon; 1966.
8. Fishman GS. Monte Carlo: concepts, algorithms, and applications. Springer; 1996.
9. Madras N. Lectures on Monte Carlo methods. Fields Institute Monographs. American Mathematical Society; 2002.
