# Supplementary material for: Calibrated simplex-mapping classification
Source: PLoS One. 2023 Jan 17;18(1):e0279876. doi: 10.1371/journal.pone.0279876 (PMC9844900; doi:10.1371/journal.pone.0279876)
Supplement: S2 Appendix — Description of the Python implementation of the proposed method. (PDF) [file pone.0279876.s002.pdf]

B Implementation

A Python implementation of our proposed method is available online [1]. The implementation includes the following main functionality, which is outlined in Algorithm 1. A sketch of the functionality is presented in Fig 10.

- Train (line 1): Calculate the simplex vertices $p_1, \ldots, p_n$ and train the regression model based on a training data set $\mathcal{D}$, the user-defined parameters α, β, k_α, k_β, and d for the training data transformation f and the hyperparameters h of the regression model. In this manner, obtain a point prediction $\widehat{f}(x)$ – possibly along with a probabilistic prediction $\widehat{q}(\,\cdot\,|x)$ – for every $x \in \mathcal{X}$. For the calculation of the simplex vertices $p_1, \ldots, p_n$, we provide two methods. First, the explicit method via (66) and second, an iterative method. For the latter, we choose p_1 to be the first canonical basis vector and determine the remaining elements of $p_2, \ldots, p_n$ one after another based on the already specified elements such that (58.b) and (68.b) are fulfilled. The resulting simplex vectors from the two methods differ only by a joint rotation so that there is no substancial effect on our method apart from minor numerical differences. All results in this paper are based on simplex vertices from the iterative method.
- PredictClassLabel (line 7): Predict the class label of (possibly unseen) feature space points $x \in \mathcal{X}$ based on the point prediction $\widehat{f}(x)$ of the trained regression model and on the simplex vertices $p_1, \ldots, p_n$.
- PredictClassLabelProbability (line 13): Predict the probability of class label $y \in \mathcal{Y}$ for unseen feature space points $x \in \mathcal{X}$ based on the probabilistic prediction $\widehat{q}(\,\cdot\,|x)$ of the trained regression model, on the simplex vertices $p_1, \ldots, p_n$, and on a fixed sample size N. We presume that $\widehat{q}(\,\cdot\,|x)$ is normally distributed with mean $\widehat{\mu}(x)$ and variance $\widehat{\sigma}(x)^2$.
- Compress (line 24): Transform the latent space point $z \in \mathcal{Z}$ to reference-simplex counterpart $w = C(z) \in -\mathcal{S}^\circ$ based on the user-defined parameter τ and the simplex vertices $p_1, \ldots, p_n$.
- Inflate (line 28): Transform the reference-simplex point $w \in -\mathcal{S}^\circ$ to latent space counterpart $z = I(w) \in \mathcal{Z}$ based on the user-defined parameter τ and the simplex vertices $p_1, \ldots, p_n$.

In particular, our implementation is intended to be used with scikit-sklearn [2]. We therefore do not prescribe the specific form of the regression model, but instead allow the user to plug in an arbitrary scikit-sklearn estimator.

References

1. Heese R. CASIMAC: Calibrated simplex mapping classifier in Python; 2022. Available online: `https://github.com/raoulheese/casimac`.
2. Pedregosa F, Varoquaux G, Gramfort A, Michel V, Thirion B, Grisel O, et al. Scikit-learn: Machine Learning in Python. Journal of Machine Learning Research. 2011;12:2825–2830.

Algorithm 1 Summary of the main functionality of the provided implementation [1].

function TRAIN($\mathcal{D}$, α, β, k_α, k_β, d, h)
    Calculate simplex vertices $p_1, \ldots, p_n$ either explicitly according to (66) using (65) and (67) or iteratively as described in appendix S2.
    Calculate $f(x_i) = f_{\alpha,\beta,k_\alpha,k_\beta,d}(x_i|\mathcal{D})$ for $i \in \{1, \ldots, D\}$ using (14) to (18)
    Calculate the transformed training data set $\mathcal{D}^f$ from (23)
    Train a regression model with hyperparameters h, by fitting it to $\mathcal{D}^f$
end function
function PREDICTCLASSLABEL(x, $\widehat{f}$, $p_1, \ldots, p_n$)
    Calculate the regression model's point prediction $\widehat{z} = \widehat{f}(x)$ for x
    Calculate the distances $\|\widehat{z} - p_1\|_2, \ldots, \|\widehat{z} - p_n\|_2$
    Calculate the label $\widehat{y} = \widehat{g}(\widehat{z})$ based on the above distances and (32)
    return $\widehat{y}$
end function
function PREDICTCLASSLABELPROBABILITY(x, y, $\widehat{\mu}$, $\widehat{\sigma}$, $p_1, \ldots, p_n$, N)
    if $n = 2$ **then**
        Calculate $\widehat{p} = \widehat{p}(y|x)$ according to (118)
    else
        Sample $z_1, \ldots, z_N$ independently from $\widehat{q}(\,\cdot\,|x)$
        Calculate the distances $\|z_i - p_1\|_2, \ldots, \|z_i - p_n\|_2$ for $i \in \{1, \ldots, N\}$
        Calculate $I_{\mathcal{C}_y}(z_1), \ldots, I_{\mathcal{C}_y}(z_N)$ based on the above distances and on (89)
        Calculate $\widehat{p} = \widehat{p}_N(y|x)$ according to (119)
    end if
    return $\widehat{p}$
end function
function COMPRESS(z, τ, $p_1, \ldots, p_n$)
    Calculate $w = C(z)$ according to (130)
    return w
end function
function INFLATE(w, τ, $p_1, \ldots, p_n$)
    Calculate $z = I(w)$ according to (131)
    return z
end function

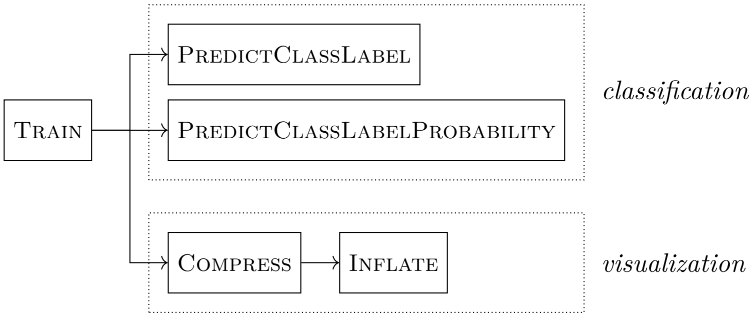

Figure 10. An overview of the functionality of the provided implementation [1] as outlined in Algorithm 1. After the initial training stage, there are two possible use cases, namely classification and visualization, as described in detail in Section 2. The first use case refers to the prediction of class labels and of class probabilities for unseen feature space points, whereas the second use case allows to perform a transformation from the latent space to a reference simplex and back for visualization purposes.
