# Supplementary material for: Calibrated simplex-mapping classification
Source: PLoS One. 2023 Jan 17;18(1):e0279876. doi: 10.1371/journal.pone.0279876 (PMC9844900; doi:10.1371/journal.pone.0279876)
Supplement: S3 Appendix — Description of the hyperparameters for the numerical benchmarks. (PDF) [file pone.0279876.s003.pdf]

Table 7. The best cross-validated hyperparameters for the real-world data sets from Table 2.

	alcohol	climate	hiv	pine	wifi
**CASIMAC**	$\gamma = 0$<br>$k_\alpha = 5$<br>$k_\beta = 5$<br>$\nu = \infty$	$\gamma = 0$<br>$k_\alpha = 5$<br>$k_\beta = 5$<br>$\nu = \infty$	$\gamma = 0$<br>$k_\alpha = 20$<br>$k_\beta = 20$<br>$\nu = \frac{3}{2}$	$\gamma = 1$<br>$k_\alpha = 1$<br>$k_\beta = 5$<br>$\nu = \frac{3}{2}$	$\gamma = 1/3$<br>$k_\alpha = 20$<br>$k_\beta = 20$<br>$\nu = \infty$
**GPC**	$\nu = \frac{3}{2}$	$\nu = \frac{3}{2}$	$\nu = \infty$	$\nu = \frac{3}{2}$	$\nu = \frac{3}{2}$
**kNN**	$k = 5$	$k = 5$	$k = 5$	$k = 5$	$k = 5$
**MLP**	$L =$<br>$(10, 10, 10)$	$L =$<br>$(5, 5)$	$L =$<br>$(5, 10)$	$L =$<br>$(10, 10, 10)$	$L =$<br>$(5, 5)$

C Hyperparameters

In Section 3 we use cross-validation to select the best hyperparameters for the classifiers out of a pre-defined set of possible choices. Specifically, we vary the number of nearest neighbors $k \in \{5, 10, 15, 20\}$ for kNN, the hidden layer sizes $L \in \{(5), (10), (5, 5), (5, 10), (10, 10), (5, 5, 5), (5, 5, 10), (5, 10, 10), (10, 10, 10)\}$ (all with a rectified linear unit as their activation function) for MLP and the kernel parameters for both GPC and the GPR model used for CASIMAC. We choose a sum of a Matérn kernel and a white-noise kernel [1] for these kernels and tune the Matérn coefficient $\nu \in \{\frac{3}{2}, \frac{5}{2}, \infty\}$. The remaining kernel parameters are optimized for each cross-validation setup as described in [1]. Additionally, for CASIMAC, we tune both γ from (43) as well as k_α and k_β according to (18). In Table 7, we list the cross-validated hyperparameters which result in the best accuracy over all classification tasks for each data set from Section 3.2. Analogously, for the synthetic data set from Section 3.1 we get the best hyperparameters $\gamma = 1$, $k_\alpha = 10$, $k_\beta = 10$, and $\nu = \infty$ for CASIMAC and $\nu = \frac{3}{2}$ for GPC. And finally, for the `alcohol-3` data set from Section 3.3 $\gamma = \frac{1}{2}$, $k_\alpha = 1$, and $k_\beta = 5$ are fixed and we find $\nu = \infty$ for CASIMAC from the cross-validation.

References

1. Rasmussen CE, Williams CKI. Gaussian Processes for Machine Learning. Adaptative computation and machine learning series. University Press Group Limited; 2006.
